# Spray-Pyrolyzed Hollow and Yolk–Shell CeO_2_ Nanocarriers with Tunable Structure for Redox-Responsive Delivery and Gene Rescue Applications

**DOI:** 10.1021/acsami.6c07497

**Published:** 2026-06-26

**Authors:** Jalal Poostforooshan, Cléa Chesneau, Laurent Michely, Madara Dias Wickramanayaka, Iwona Pranke, Patrick Balaguer, Abdelhay Boulahtouf, Fabrice Lejeune, Benedikt Eberhardt, Alexandre Hinzpeter, Sabrina Belbekhouche, Alfred P. Weber

**Affiliations:** † 129409 Institute of Particle Technology, Clausthal University of Technology, 38678 Clausthal-Zellerfeld, Germany; ‡ Univ Paris Est Creteil, CNRS, Institut de Chimie et des Matériaux Paris-Est (ICMPE), UMR 7182, 2 Rue Henri Dunant, 94320 Thiais, France; § 555089 Université Paris Cité, CNRS, UMR8253, INSERM, U1151, Institut Necker Enfants Malades (INEM), F-75015 Paris, France; ∥ Institut de Recherche en Cancérologie de Montpellier (IRCM), Inserm, U1194, Université de Montpellier, Institut régional du Cancer de Montpellier (ICM), 34090 Montpellier, France; ⊥ Univ. Lille, Inserm, CHU Lille, CNRS, U1366-UMR9020, CRCLille, Cancer Research Center of Lille, F-59000 Lille, France

**Keywords:** mesoporous CeO_2_ nanoparticles, spray pyrolysis, hollow and yolk−shell structures, redox-responsive nanocarriers, translational readthrough, CFTR-R553X gene rescue

## Abstract

Mesoporous CeO_2_ nanoparticles (MCNs) are promising for biomedical applications because of their intrinsic redox activity and favorable biocompatibility. In particular, MCNs with hollow and yolk–shell nanostructures have attracted considerable interest as nanocarriers due to their large internal volume, tunable shell porosity, and ability to regulate mass transport. However, the synthesis of hollow and yolk–shell MCNs remains challenging using conventional solution-based methods. Here, we report a versatile gas-phase spray pyrolysis approach for the synthesis of MCNs with tunable structures via atomization of cerium nitrate solutions in the presence of polyvinylpyrrolidone (PVP) as a structure-directing agent. By systematically varying precursor composition, droplet size, and thermal processing conditions, the internal cavity formation, particle morphology, and mesoporous structure were precisely controlled. A custom-designed multizone furnace further enabled stepwise observation of particle formation, providing mechanistic insights into the morphological evolution during spray pyrolysis. Moreover, surface functionalization of MCNs with thiolated dextran introduced redox-responsive behavior through disulfide linkages that are cleavable under intracellular reductive conditions. Biological evaluation demonstrated excellent biocompatibility in HEK293 cells and revealed that the Rhodamine 6G loading efficiency strongly correlates with internal void volume and particle morphology. Furthermore, the functionalized MCNs significantly enhanced the efficacy of the aminoglycoside antibiotic G418, promoting translational readthrough in U2OS reporter cells and restoring CFTR-R553X activity in epithelial monolayers. These findings highlight spray-pyrolyzed mesoporous CeO_2_ hollow and yolk–shell nanoparticles as promising redox-responsive nanocarriers for intracellular delivery and therapeutic modulation of genes affected by nonsense mutations.

## Graphical abstract

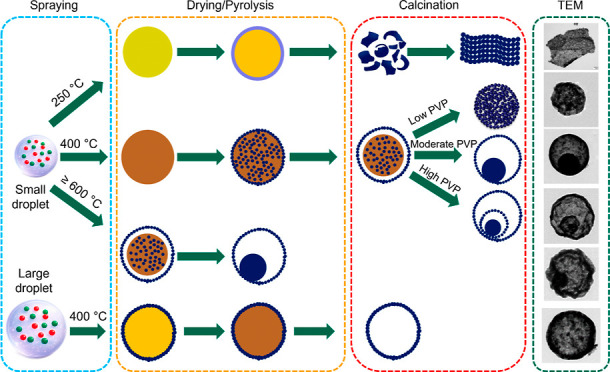

## 1 Introduction

Cerium dioxide (CeO_2_) has attracted considerable attention due to its high oxygen storage capacity, thermal stability, and excellent biocompatibility.^1–4^ Its unique redox properties originate from the reversible transition between Ce^3+^ and Ce^4+^ oxidation states, accompanied by the formation of oxygen vacancies within the fluorite crystal structure.^5^ The performance of CeO_2_ is strongly influenced not only by defect chemistry, particularly Ce^3+^ ions and oxygen vacancies, but also by structural parameters such as surface area, porosity, and particle morphology.^6–9^ Tailoring these structural features enhances active site accessibility, facilitates mass transport, and improves stability under operating conditions. Therefore, various nanostructured CeO_2_ morphologies, such as solid, core–shell, hollow, and yolk–shell structures, have been developed to improve performance for diverse applications.^10–12^ Among these nanostructures, hollow and yolk–shell nanoparticles have gained considerable attention due to their internal cavities and tunable shell structures, offering multiple functional advantages. Hollow particles offer low density and a high loading capacity that can accommodate guest molecules. Yolk–shell structures represent a more advanced configuration in which a movable core is confined within a hollow shell separated by a void space. This core@void@shell architecture provides several advantages, including enhanced structural stability, improved accessibility of active surfaces, and controlled diffusion through the mesoporous shell. In addition, the presence of an inner core within the hollow cavity can promote multiple internal light reflections, which is advantageous for photon harvesting.^13–16^ Such structural features make hollow and yolk–shell structures particularly attractive as versatile platforms for applications ranging from catalysis and energy storage to drug delivery and nanomedicine.^17–19^

Despite these advantages, the synthesis of hollow and yolk–shell nanostructures remains challenging. Most reported methods rely on solution-based, template-assisted techniques, such as sol–gel, precipitation, and microemulsion approaches.^14,20^ These methods typically involve the formation of a core–shell intermediate with a sacrificial inner layer, followed by selective removal of the inner layer to create the internal void. However, such methods are often limited by complex multistep procedures, long processing times, and the need for strong acids or alkaline solutions as etching agents, leading to environmental pollution.^21–24^ Furthermore, the removal of the template may cause partial collapse of the shell.^25,26^ These techniques also often require highly diluted precursor solutions to ensure uniform coating and minimize particle aggregation, resulting in low production yields and increased energy consumption during particle collection.^27^

To overcome these drawbacks, a few studies have explored the fabrication of yolk–shell nanostructures via spray pyrolysis, a promising continuous synthesis method.^28–32^ Compared to traditional solution-based methods, spray pyrolysis has several distinct advantages, including rapid synthesis, process simplicity, and scalability.^33^ This method eliminates the need for multiple templating, washing, or etching steps and enables direct collection of dry powders. Moreover, each atomized droplet behaves as an individual microreactor as it passes through the furnace, significantly reducing particle aggregation and ensuring uniform particle formation.^12,34^ More importantly, the internal cavity structure, particle morphology, and porosity of the mesoporous shell can be finely tuned by adjusting parameters such as precursor composition, droplet size, and thermal conditions, allowing precise control over loading capacity and controlled-release properties. In this work, we present, for the first time, a gas-phase synthesis route for producing mesoporous CeO_2_ nanoparticles (MCNs) with tailored hollow and yolk–shell architectures via spray pyrolysis of cerium nitrate (CeN) in the presence of polyvinylpyrrolidone (PVP) as a structure-directing agent. By systematically varying synthesis parameters, we investigate the relationship between processing conditions and the resulting structural features, enabling precise control over internal cavity formation, shell morphology, and mesoporosity.

Beyond structural design, the incorporation of responsive surface chemistry is essential for enabling advanced biomedical functionality.^35^ Hollow and yolk–shell CeO_2_ nanostructures are particularly attractive as nanocarriers because their internal cavities provide large storage volumes for therapeutic molecules, while mesoporous shells regulate diffusion and release behavior.^1^ To further enhance their performance in biological environments, surface functionalization with redox-responsive polymers can provide stimuli-triggered release mechanisms and improved colloidal stability. Dextran is a biocompatible polysaccharide widely used in biomedical applications due to its hydrophilicity, low immunogenicity, and ease of chemical modification.^36^ In particular, thiolated dextran introduces disulfide bonds that are stable under extracellular conditions but cleavable in the reductive intracellular environment, where glutathione concentrations are significantly higher.^37^ This redox-responsive behavior provides an efficient strategy for triggered cargo release inside cells while minimizing off-target effects. Functionalizing mesoporous CeO_2_ nanostructures with thiolated dextran is therefore expected to improve colloidal stability in biological media, reduce nonspecific interactions with biological components, and enable intracellular, reduction-triggered release of loaded probes or drugs. In this study, the biological performance of dextran-functionalized MCNs was evaluated through a series of in vitro experiments, including cytotoxicity assessment, probe loading capacity, and redox-responsive release behavior.

Finally, the potential of these nanocarriers for intracellular delivery was investigated in a functional biological context by evaluating their ability to enhance G418-induced translational readthrough of premature stop codons. Translational readthrough is a natural mechanism by which the ribosome bypasses a premature stop codon and continues translation of the mRNA (PMID: 33089614; PMID: 33751142). Aminoglycoside antibiotics such as G418 are known to promote readthrough of nonsense mutations, restoring partial expression of full-length proteins.^38^ In particular, the rescue of cystic fibrosis transmembrane conductance regulator (CFTR) function in cells harboring the CFTR-R553X nonsense mutation represents a clinically relevant model for cystic fibrosis. By improving intracellular delivery and local concentration of G418, the developed CeO_2_-based nanocarriers aim to enhance translational readthrough efficiency and restore functional CFTR expression in human cells, highlighting their potential as a platform for therapeutic delivery in genetic diseases caused by nonsense mutations.

## 2 Experiment

### 2.1 Materials

Cerium­(III) nitrate hexahydrate (Ce­(NO_3_)_3_·6H_2_O, ≥99% purity), polyvinylpyrrolidone (PVP, average molecular weight ≈ 10,000), hydrogen peroxide (30%), ammonia solution (∼1 M NH_3_ in H_2_O), ethanol (96%), (3-glycidoxypropyl)­trimethoxysilane, 2-aminoethanethiol, chloramine T, rhodamine 6G, and glutathione were obtained from Sigma-Aldrich. Thiolated dextran (Dex6-SH) was synthesized according to the procedure reported by Chesneau et al.^39^ Deionized water was used as the solvent throughout all experiments.

### 2.2 Synthesis of Morphology-Tailored Mesoporous CeO_2_ Nanoparticles

In this work, mesoporous CeO_2_ nanoparticles (MCNs) with tunable morphologies were synthesized via spray pyrolysis. In a typical procedure, 5 g of Ce­(NO_3_)_3_·6H_2_O (CeN) and a specified amount of polyvinylpyrrolidone (PVP, structure-directing agent) were dissolved in 100 mL of deionized water to obtain PVP-to-Ce mass ratios of 0.25, 0.5, 1, and 2. The resulting precursor solution was atomized using a nebulizer, with compressed air as the carrier gas. The generated aerosol was passed through a single-zone quartz tube furnace (inner diameter: 26 mm; length: 650 mm) maintained at 400 °C. The as-synthesized particles were collected directly on filter paper and subsequently calcined in air at 500 °C for 6 h (heating rate: 3 °C/min) to remove residual organics and enhance crystallinity. To evaluate the role of droplet size on particle morphology, two types of nebulizers were employed: a Topas ATM 220, generating smaller droplets, and a Palas AGK-2000, producing larger droplets. For the Topas system, an airflow of 2 L/min corresponded to an aerosol residence time of ∼310 ms, whereas the Palas system operated at 4.2 L/min, yielding a residence time of ∼130 ms. In a separate series of experiments, the effect of furnace temperature on particle formation was investigated using the Topas nebulizer and a fixed PVP-to-Ce mass ratio of 2. The temperature of the single-zone furnace was systematically varied from 250 to 1000 °C, while all other parameters were kept constant. For clarity, the synthesized and calcined samples are denoted as x-MCN-y-z-C, where x denotes the nebulizer type (S for Topas, L for Palas), y the furnace temperature in °C, z the PVP-to-Ce mass ratio, and C indicates that the sample was calcined.

### 2.3 Investigation of Morphological Transitions via Multi-Zone Spray Pyrolysis

To gain a better understanding of the formation process of yolk–shell-structured MCNs, a custom-built three-zone tube furnace was employed, with sequential zone temperatures set to 250 °C, 400 °C, and 600 °C. A precursor solution with a fixed PVP-to-Ce mass ratio of 0.5 was atomized using the Topas ATM 220 nebulizer and processed under identical spray pyrolysis conditions as described above. Particles were collected separately at dedicated outlets between heating zones, enabling sampling after each temperature stage to track the morphological transitions during the spray pyrolysis process. Details of the three-zone furnace configuration are provided in the Supporting Information (see Section S1). The synthesized samples are denoted as MZ-MCN-y-0.5-C, where MZ refers to the multizone setup, y indicates the zone temperature (°C), 0.5 represents the PVP-to-Ce mass ratio, and C shows postcalcination.

### 2.4 Surface Functionalization of MCNs with Thiolated Dextran

A total of 100 mg of CeO_2_ particles were first treated in an oxidizing basic medium composed of 0.5 mL H_2_O_2_ and 0.5 mL NH_4_OH for 1 h to increase the density of surface hydroxyl groups. After this hydroxylation step, the particles were collected by centrifugation at 6000 rpm for 15 min, washed twice with distilled water, and once with ethanol. The particles were then dispersed in 10 mL of ethanol containing 100 μL of 3-glycidoxypropyltrimethoxysilane and stirred for 3 h to introduce a silane layer on the surface. Subsequently, 2-aminoethanethiol (16 mg in 2 mL ethanol) was added to functionalize the terminal epoxy groups and incorporate thiol moieties. The suspension was stirred for 24 h, followed by centrifugation and sequential washes with ethanol (two times) and 0.1 M PBS (pH 7.4). For the final grafting step, the thiolated dextran derivative Dex6-SH was coupled to the modified particle surface using chloramine T as an oxidizing agent. A mixture of 10 mg Dex6-SH and 3.5 mg chloramine T in 1 mL of 0.1 M PBS (pH 7.4) was added to the particles and gently stirred for 20 min to promote disulfide bond formation. The functionalized MCNs were then collected by centrifugation and dried at 80 °C for 24 h.

### 2.5 Sample Characterization

The morphology and structure of the synthesized MCNs were analyzed using transmission electron microscopy (TEM, JEOL JEM-2100, operating at 160 kV) and scanning electron microscopy (SEM, Zeiss DSM Gemini 982, operated at 5 kV). Nitrogen adsorption–desorption measurements were conducted using a Micromeritics ASAP 2020 analyzer. Specific surface areas were determined using the Brunauer–Emmett–Teller (BET) method, while average pore sizes were calculated from the adsorption branch of the isotherms using the Barrett–Joyner–Halenda (BJH) model. Thermal behavior was assessed by thermogravimetric analysis (TGA) using a NETZSCH TG 209 F1 instrument in an air atmosphere with a heating rate of 10 °C/min. In addition, TGA combined with mass spectral analysis of gaseous products (TGA-MS) was used to study the thermal transformation of CeN by STA 409 PC Luxx thermal analyzer with a quadrupole mass spectrometer QMS 403C Aëolos (Netzsch-Gerätebau GmbH, Selb, Germany). The CeN was heated up to 800 °C at a heating rate of 10 °C per min in air or argon. Raman spectroscopy was performed at room temperature using a WITec alpha300R confocal Raman microscope. The droplet size was measured by laser diffraction method using a Helos/BR (Sympatec). Crystalline phases were identified via X-ray diffraction (XRD) using Cu Kα radiation (λ = 1.5406 Å) with a germanium monochromator. The average crystallite size was estimated using Scherrer’s eq (eq 1).

1 $$D=\frac{k\lambda }{\beta \cos\theta }$$

where *D* is the crystallite size, *k* = 0.90 is the shape factor, λ is the X-ray wavelength, β is the full width at half-maximum (fwhm) of the selected diffraction peak (in radians), and θ is the Bragg angle. The isoelectric point of the MCNs was determined by measuring the zeta potential of aqueous suspensions as a function of pH over the range 2–12. The particles were dispersed in distilled water at a concentration of 0.1 g·L^–1^, and the pH was adjusted using 0.1 M HCl or 0.1 M NaOH. The suspensions were vortex-mixed to ensure adequate homogenization prior to analysis. Zeta potential measurements were performed in triplicate using a dynamic light scattering instrument (Zetasizer Nano ZS, Malvern Instruments, USA).

To investigate the interaction between MCNs and rhodamine 6G (Rh6G), a positively charged fluorescent dye, samples were prepared under pH conditions that rendered the MCNs negatively charged. Under these conditions, identified in this study as favoring electrostatic binding, the cationic dye is expected to adsorb onto the particle surfaces. An aqueous solution of Rh6G (1 g·L^–1^, pH 7.4) was mixed with 10 mg of MCNs and stirred for 24 h. After incubation, the particles were collected by centrifugation and washed repeatedly with water to remove any unadsorbed dye. The encapsulation efficiency was quantified by measuring the absorbance of the supernatants at 525 nm using UV–visible spectroscopy, and the remaining dye concentration was determined from a calibration curve established with Rh6G (ε exp = 96,012 L·mol^–1^·cm^–1^). The entrapment efficiency was then calculated using eq 2.

2 $$Entrapment\;efficiency\left(\%\right)=\frac{mass\;of\;entrapped\;probe\;in\;final\;product}{initial\;mass\;of\;probe}\times 100$$

Additionally, the loading of Rh6G on the MCNs was examined using a ZEISS fluorescence optical microscope.

### 2.6 Biological Assay

#### 2.6.1 Cell Culture

HEK293 cells were cultured in Dulbecco’s Modified Eagle Medium (DMEM) supplemented with 1% penicillin/streptomycin and 10% Fetal Bovine Serum (FBS) (all from Invitrogen). The cells were maintained at 37 °C with 5% CO_2_. U2OS cells were cultured in DMEM (Invitrogen) supplemented with 1% Zellshield (Minerva) and 10% FBS. The cells were maintained at 37 °C with 5% CO_2_. 16HBEo-WT and genetically engineered 16HBEo-R553X (PMID:30563749) cells were cultured in Minimum Essential Medium (MEM, Invitrogen) supplemented with 10% FBS and 1% Penicillin/Streptomycin. Both cell lines were cultured in fibronectin-coated T75 flasks and maintained at 37 °C with 5% CO_2_. Cells were seeded at a density of 1 × 10^5^ cells per filter onto fibronectin-coated microporous filters (0.33 cm^2^) in a 24-well plate system (Corning Inc., Corning, NY, USA), under submerged culture conditions. The cells were allowed to polarize under liquid–liquid conditions for a week with medium changes every 2 days, with 300 μL in the apical side and 700 μL in the basolateral side.

#### 2.6.2 Toxicity Assay

For cell viability assays, HEK293 cells were seeded in a 96-well plate and cultured to achieve 80% confluence after 24 h. The following day, cells were incubated for 24 h in complete media and different concentrations of nanoparticles that had been resuspended in water. Cytotoxicity was evaluated through photocolorimetric analysis using the Cell Counting Kit 8 (WST-8) according to the manufacturer’s guidelines (Abcam, Ab228554). This involved diluting CCK8 in DMEM with FBS (1:10 ratio) and adding 100 μL to each well. The cells were then incubated for 1 h in a 37 °C, 5% CO_2_ environment. Absorbance was measured using a ClarioStar plate reader (BMG) at 460 nm. Each measurement was conducted in duplicate across at least five independent experiments. Results are expressed as a percentage of the untreated control while a 1% SDS solution served as a positive control.

#### 2.6.3 Readthrough Assay Using an In Vitro Luciferase Reporter Assay

U2OS cells stably express a luciferase cassette harboring a UGA premature termination codon (PTC). PTC-readthrough inducer G418 (Invitrogen) was diluted in PBS and incubated 30 min at room temperature with 100 μg/mL of the considered nanoparticle. Cells were seeded in 96-well plates and incubated 24 h with G418 alone or complexed with the nanoparticle. Luciferase activity was measured on a plate reader (TriStar, Berthold). Each measurement was conducted across at least three independent experiments.

#### 2.6.4 Multi Transepithelial Clamp Current

One week after seeding on filters, the cells were treated for 48 h with a concentration range of G418 (Invitrogen) from 50 to 400 μg/mL without or with a 30 min preincubation with 100 μg/mL of nanoparticles. To maximize CFTR amounts, cells were incubated with CFTR correctors VX-445 (3 μM) and VX-809 (3 μM) (Selleckchem) and nonsense-mediated decay inhibitor SMG1 (0.5 μM) (Sigma-Aldrich). Cell culture electrophysiological properties were measured using a gradient assay MTECC. Transepithelial electrical resistance (*R*_t_) and open-circuit potential difference (PD) of the epithelial monolayers were recorded using electrodes positioned in close contact with the filters and controlled by a robotic system. The medium was changed right before measurements with two buffers (pH 7.4) containing KCl (4 mM), CaCl_2_ (1.8 mM), MgCl_2_ (1 mM), HEPES (10 mM), d-Glucose (10 mM), the one in the basolateral side has NaCl (137 mM) and the one in the apical side has Na-Gluconate (137 mM) (all from Sigma-Aldrich). Cells were maintained at 37 °C and oxygenated with ambient air.

Once the baseline current was established, apical treatment with agonists and inhibitors were added sequentially according to a standard Ussing chamber-like protocol. First, cAMP agonists Forskolin (10 μM Sigma-Aldrich) and 3-isobutyl-1-methylxanthine (IBMX, 100 μM, Sigma-Aldrich) to activate the transepithelial cAMP-dependent current (including Cl- transport through CFTR channels); VX-770 (1 μM to potentiate CFTR channel, Selleckchem); CFTR inhibitor inh172 (5 μM, Sigma-Aldrich) to specifically inhibit CFTR. *R*_t_ and PD were used to calculate the value of current (Ieq) using Ohm’s Low. Ieq change after CFTR inhibition by Inh-172 served as an index of CFTR function. Only samples with a resistance >200 Ω·cm^2^ were analyzed.

## 3 Results and Discussion

### 3.1 Synthesis and Morphological Tailoring of MCNs

The goal of this study was to develop mesoporous CeO_2_ nanoparticles (MCNs) with tunable structural features for cargo-delivery applications. We hypothesized that key physicochemical properties of these particles, including internal structure, morphology, porosity, and crystallinity, are significantly influenced by spray pyrolysis conditions. To this end, a series of systematic experiments was carried out. MCNs were synthesized via spray pyrolysis of aqueous precursor solutions containing CeN and PVP, followed by calcination to remove the PVP template. The influence of PVP-to-Ce mass ratio on the evolution of yolk–shell structures was first examined, after which the roles of droplet size and furnace temperature profile were studied using both single- and multizone furnace configurations.

#### 3.1.1 Effect of PVP-to-Ce Mass Ratios on MCNs Formation

The influence of the PVP-to-Ce mass ratio on the internal structure of MCNs nanoparticles was systematically investigated. Figure 1 presents the corresponding TEM images of particles synthesized at 400 °C using the Topas atomizer. It was observed that at a low mass ratio of 0.25 (S-MCN-400–0.25-C), the particles exhibit incomplete or irregular yolk–shell structures, suggesting that the amount of PVP was insufficient to induce proper phase separation during particle formation (Figure 1a). Increasing the PVP content to a mass ratio of 0.5 (S-MCN-400–0.5-C) resulted in the formation of well-defined yolk–shell structures, with a distinct hollow space between the dense core and the porous shell (Figure 1b). The shell appears to be composed of aggregated CeO_2_ nanocrystals, forming a mesoporous framework. Further increasing the PVP-to-Ce mass ratio to 1 (S-MCN-400–1-C) led to yolk–shell particles with smaller cores (Figure 1c). Interestingly, at a high PVP-to-Ce mass ratio of 2 (S-MCN-400–2-C), most particles exhibited a double-shelled yolk–shell morphology (Figure 1d). This observation suggests that the excess amount of PVP may promote the formation of secondary shells, likely driven by enhanced phase separation or the influence of carbonaceous intermediates formed during thermal treatment. These results confirm that the PVP-to-Ce mass ratio is a key parameter for controlling the internal design of yolk–shell-structured MCNs. TEM-based particle-size analysis was performed using ImageJ software to confirm the particle size of the synthesized MCNs. The mean particle diameters were 146 ± 74, 207 ± 137, 228 ± 140, and 206 ± 120 nm for S-MCN-400–0.25-C, S-MCN-400–0.5-C, S-MCN-400–1-C, and S-MCN-400–2-C, respectively.

**Figure 1:**
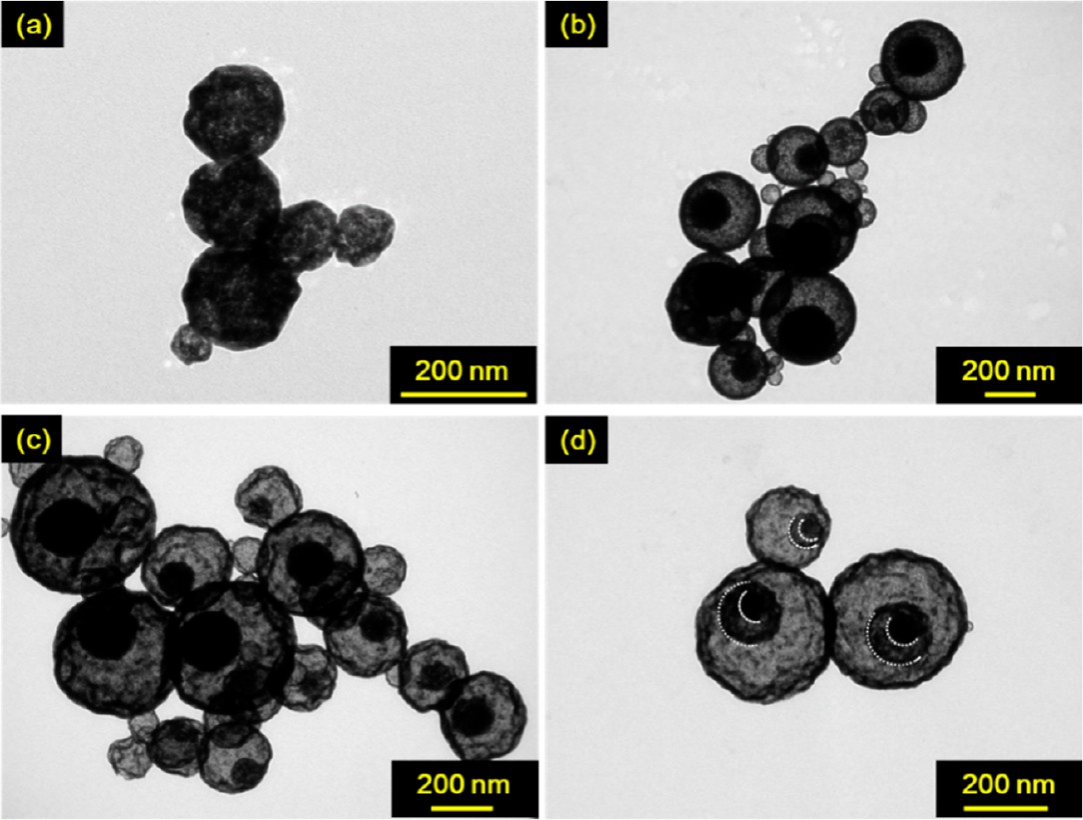
TEM images of (a) S-MCN-400–0.25-C, (b) S-MCN-400–0.5-C, (c) S-MCN-400–1-C, and (d) S-MCN-400–2-C particles prepared using spray pyrolysis.

To quantitatively evaluate the influence of the PVP content on the internal structure of the yolk–shell-structured MCNs, the void volume between the yolk and the shell was calculated based on geometric parameters extracted from TEM images using the image analysis software ImageJ. The overall particle diameter, shell thickness, and yolk diameter were measured for multiple particles from different TEM images, and the averaged values were used for the void-volume calculation. The void volume V was determined using eq 3.^40^

3 $$V=\left(4/3\right)\cdot \pi \cdot \left[{\left(D/2-S\right)}^{3}-{\left(Y/2\right)}^{3}\right]$$

where *D* is the overall outer diameter of the well-defined yolk–shell particle, *S* is the shell thickness, and *Y* is the yolk diameter. The specific values used for each parameter are provided in Table S1. For S-MCN-400–2-C particles with a double-shell morphology, the total void space was obtained by summing the two separate voids (eq S2): the first between the yolk and the inner shell, and the second between the inner shell and the outer shell. The calculated void volumes for S-MCN-400–0.5-C, S-MCN-400–1-C, and S-MCN-400–2-C were approximately 1.78 × 10^7^ nm^3^, 1.87 × 10^7^ nm^3^, and 0.96 × 10^7^ nm^3^, respectively. These results reveal that while increasing the PVP content initially enhances the void space by reducing the yolk size, further increases lead to structural transformations such as double-shell formation with lower internal free volume. To assess how these structural variations influence functional performance, the probe loading capacity was evaluated using rhodamine 6G (Rh6G) as a model molecule (see Section 3.4.2 and Figure 12), enabling a direct correlation between internal structure and loading behavior.

The surface morphologies of the synthesized MCNs are presented in Figure S1. At the low ratio of 0.25, the particles exhibit irregular and agglomerated structures (Figure S1a), indicating insufficient PVP to effectively form spherical particles. As the ratio increases to 0.5 and 1 (Figure S1b,c), more uniform and spherical particles form, indicating that moderate PVP content improves templating and shell formation. Furthermore, at the highest ratio of 2 (Figure S1d), the particles maintain their spherical shape but exhibit wrinkled surfaces, likely due to structural rearrangements during calcination.

To determine the amount of PVP template incorporated into the as-synthesized MCNs, thermogravimetric analysis (TGA) was performed on the composite powders prior to calcination (Figure 2a). Pure PVP exhibited a major weight loss between 300 and 500 °C, corresponding to its thermal decomposition.^41^ Similarly, all composite samples showed significant weight losses in the 250–500 °C range, attributed to the decomposition of the PVP template. Based on the TGA results, the weight percentage of PVP template in the composites with mass ratios of 0.25, 0.5, 1, and 2 was approximately 17%, 38%, 58%, and 74%, respectively. In this method, during the spray pyrolysis, spherical PVP-CeO_2_ composite particles were initially formed. Subsequent calcination led to the thermal removal of PVP, resulting in the development of yolk–shell-structured CeO_2_ particles, as the mechanism will be discussed in more detail in the following sections. These results, together with TEM analysis, indicate that the concentration of PVP plays a crucial role in morphological evolution. At low PVP content (∼17%), yolk–shell structures were not formed, whereas at high content (∼74%), a more complex double-shell yolk–shell morphology was observed.

**Figure 2:**
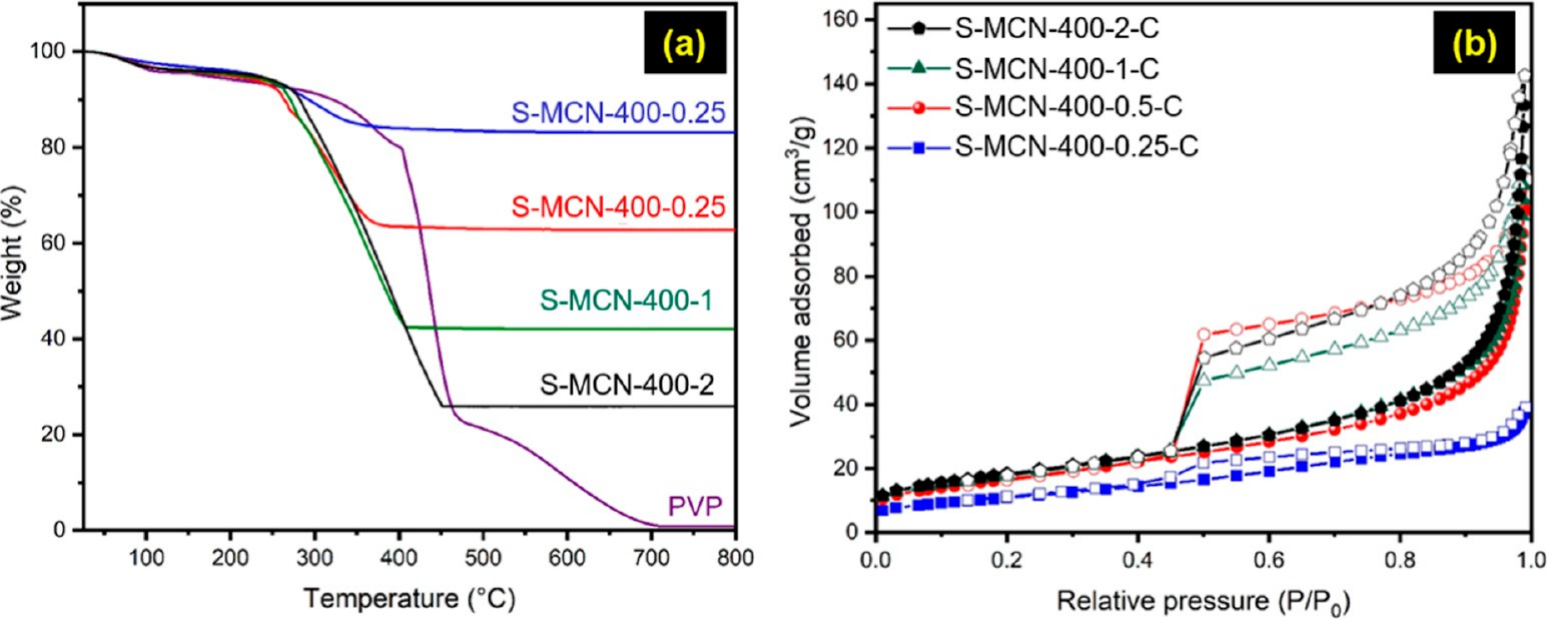
(a) Thermogravimetric curves of the as-synthesized CeO_2_ particles with different mass ratios of PVP-to-Ce and (b) N_2_ adsorption–desorption isotherms of MCNs.

The textural characteristics of the synthesized yolk–shell-structured MCNs were investigated using nitrogen adsorption–desorption isotherms (Figure 2b). Samples prepared with PVP-to-Ce mass ratios of 0.5, 1, and 2 exhibit a typical type IV isotherm with an H_2_-shaped hysteresis loop, which confirms the successful formation of mesoporous shells within the yolk–shell structure.^42,43^ In contrast, the sample with the lowest PVP content (S-MCN-400–0.25-C) shows a type IV isotherm with an H_3_-type hysteresis loop, typically associated with slit-like pores or aggregates of plate-like particles.^12,44,45^ The calculated BET specific surface areas increase with higher PVP content, with values of 39, 60, 64, and 65 m^2^/g for the 0.25, 0.5, 1, and 2 mass ratio samples, respectively. These results confirm that increasing PVP content improves the development of porosity and accessible surface area. Moreover, the BJH pore size distribution (Figure S2a) shows that the mesopores of all samples are primarily centered around 2–3 nm. The textural parameters, including BET specific surface area, dominant BJH pore diameter, and total pore volume, are summarized in Table 1.

**Table 1:** BET Surface Areas, BJH Dominant Pore Sizes, and Pore Volumes of MCN Samples

sample	BET surface area (m^2^ g^–1^)	BJH dominant pore size (nm)	total pore volume (cm^3^ g^–1^)
S-MCN-400–0.25-C	39	2.3	0.06
S-MCN-400–0.5-C	60	2.3	0.17
S-MCN-400–1-C	64	2.3	0.17
S-MCN-400–2-C	65	2.3	0.22
L-MCN-400–0.25-C	46	2.8	0.06
L-MCN-400–0.5-C	60	2.4	0.09
L-MCN-400–1-C	76	2.5	0.14
L-MCN-400–2-C	89	2.5	0.19
S-MCN-250–2-C	120	2.5	0.2
as-synthesized S-MCN-600–2	74	2.1	0.2
as-synthesized S-MCN-800–2	68	2.1	0.18
as-synthesized S-MCN-1000–2	38	2.3	0.17

X-ray diffraction (XRD) was carried out to identify the crystal structure of the synthesized MCNs (Figure S2b). All samples exhibit distinct diffraction peaks at 2θ values of 28.5°, 33.1°, 47.5°, 56.3°, 59.1°, 69.5°, 76.8°, 79.1°, and 88.4°, which can be indexed to the (111), (200), (220), (311), (222), (400), (331), (420), and (422) planes of the face-centered cubic (FCC) fluorite phase of CeO_2_.^12,46^ These peaks are in good agreement with the reference pattern of cubic CeO_2_ (JCPDS No. 34-0394), confirming the successful formation of phase-pure CeO_2_ without detectable crystalline impurities.^47^ The average crystallite sizes, calculated using Scherrer’s equation, are approximately 9 nm for all samples, indicating that variations in PVP concentration do not significantly affect crystallite growth under identical thermal treatment conditions. Together with the shell morphology revealed by TEM and the removal of the PVP template verified by TGA after calcination, these XRD results support that the shells and cores of the hollow and yolk–shell particles are composed predominantly of crystalline CeO_2_.

#### 3.1.2 Effect of Droplet Size on Particle Morphology

To study the effect of droplet size on the morphology and the internal structure of resulting particles, the Palas AGK-2000 atomizer was employed under the same spray pyrolysis conditions. As shown in Figure S3, the average droplet sizes produced by the Topas ATM 220 (Figure S3a) and Palas AGK-2000 (Figure S3b) atomizers were approximately 1.6 and 5.5 μm, respectively.

Figure 3 shows TEM images of MCNs synthesized using the Palas atomizer (L-MCN-400-z-C series) with varying PVP-to-Ce mass ratios. At the lowest mass ratio of 0.25, the particles appeared irregular (Figure 3a). Interestingly, at the intermediate and optimal mass ratio of 0.5, well-formed hollow MCNs were produced (Figure 3b). Increasing the PVP-to-Ce mass ratio to 1 resulted in larger hollow/porous particles with partially collapsed and wrinkled shell structures (Figure 3c). At the highest ratio of 2, porous film-like structures were observed (Figure 3d). These results clearly demonstrate that increasing droplet size changes the particle formation mechanism, as larger droplets tend to promote the formation of hollow structures rather than yolk–shell structures. For the L-MCN-400-z-C samples, the mean particle diameters were 140 ± 109, 159 ± 85, 231 ± 90, and 190 ± 94 nm for L-MCN-400–0.25-C, L-MCN-400–0.5-C, L-MCN-400–1-C, and L-MCN-400–2-C, respectively.

**Figure 3:**
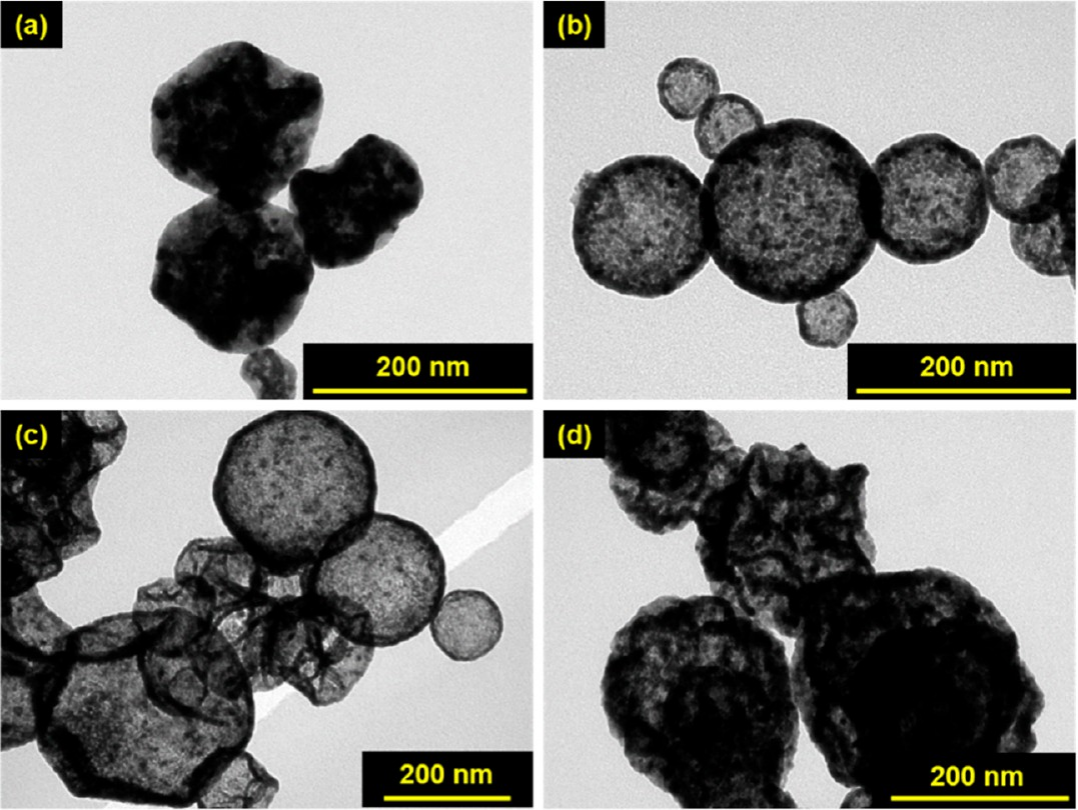
TEM images of (a) L-MCN-400–0.25-C, (b) L-MCN-400–0.5-C, (c) L-MCN-400–1-C (d) L-MCN-400–2-C particles.

TGA analysis indicates that the incorporated PVP content in the as-synthesized CeO_2_ composites is approximately 43%, 59%, 74%, and 84% for mass ratios of 0.25, 0.5, 1.0, and 2.0, respectively (Figure S4). These results confirm that the mass ratio of 0.5 is optimal for forming hollow particles with a stable structure. At higher ratios, particularly 2.0, the excess PVP (84%) leads to unstable shell formation and partial collapse during the template removal process.^48^

Figure 4a presents the N_2_ adsorption–desorption isotherms of the L-MCN-400-z-C series. All samples exhibit type IV isotherms with hysteresis loops, confirming their mesoporous structure. The hysteresis loop shape changes with PVP content. At lower ratios (0.25 and 0.5), H_2_-type loops dominate, suggesting ink-bottle-shaped pores.^49^ However, at higher ratios (1.0 and 2.0), the loops shift to H_3_-type, characteristic of slit-shaped pores typically associated with collapsed or layered structures. BET analysis shows a corresponding increase in specific surface area from 46 m^2^/g (*z* = 0.25) to 60, 76, and 89 m^2^/g for *z* = 0.5, 1.0, and 2.0, respectively (Table 1). Furthermore, the BJH pore size distributions (Figure S5) reveal narrow mesopores centered around 3–4 nm, with the total pore volume increasing with higher PVP content. XRD analysis of the L-MCN-400-z-C samples confirms that all samples possess the FCC crystal structure of CeO_2_ (Figure 4b). The average crystallite size is approximately 9 nm, suggesting that variations in droplet size have a negligible effect on crystallite growth under identical calcination conditions.

**Figure 4:**
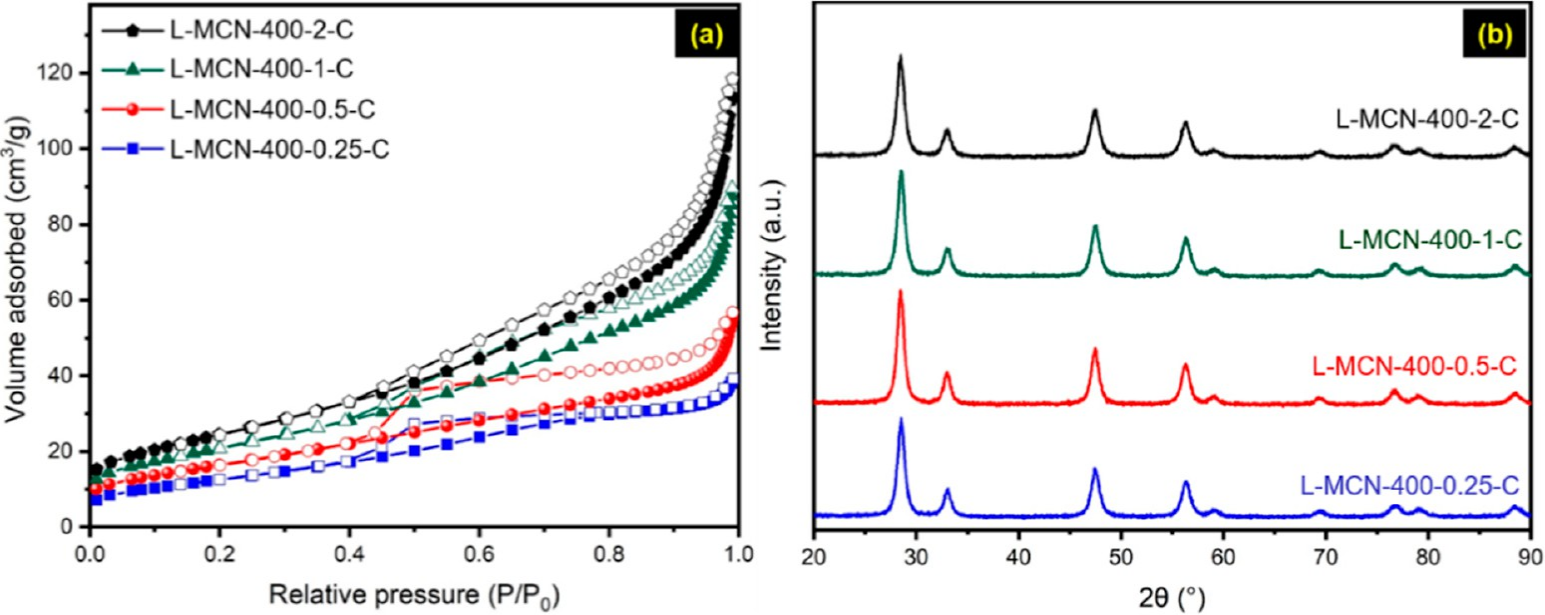
(a) N_2_ adsorption–desorption isotherms and (b) XRD patterns of L-MCN-400-z-C samples.

#### 3.1.3 Effect of Furnace Temperature on Particle Morphology

To investigate the influence of furnace temperature on particle morphology, a separate series of experiments was conducted using the Topas ATM 220 nebulizer and a fixed PVP-to-Ce mass ratio of 2.0, while keeping all other parameters constant. The furnace temperature was varied from 250 to 1000 °C. At 250 °C, the final produced MCNs after postcalcination (S-MCN-250–2-C) exhibit mesoporous nanosheet morphology, composed of interconnected nanoparticles forming sheet-like aggregates (Figure 5a). More importantly, when the synthesis temperature was increased to 600 °C, 800 °C, and 1000 °C, yolk–shell-structured MCNs formed directly during spray pyrolysis, eliminating the need for postcalcination (Figure 5b–d). It is worth noting that these particles mainly exhibit a single-shell structure, while double-shell structures only formed in the sample synthesized at 400 °C, followed by postcalcination (S-MCN-400–2-C), as shown in Figure 1d. These results demonstrate that furnace temperature strongly affects both particle formation and internal morphology by controlling the decomposition kinetics of CeN and the PVP template. This aspect will be further discussed in the mechanism section. The as-synthesized temperature-series samples S-MCN-600–2, S-MCN-800–2, and S-MCN-1000–2 showed mean particle diameters of 180 ± 100, 213 ± 103, and 170 ± 110 nm, respectively.

**Figure 5:**
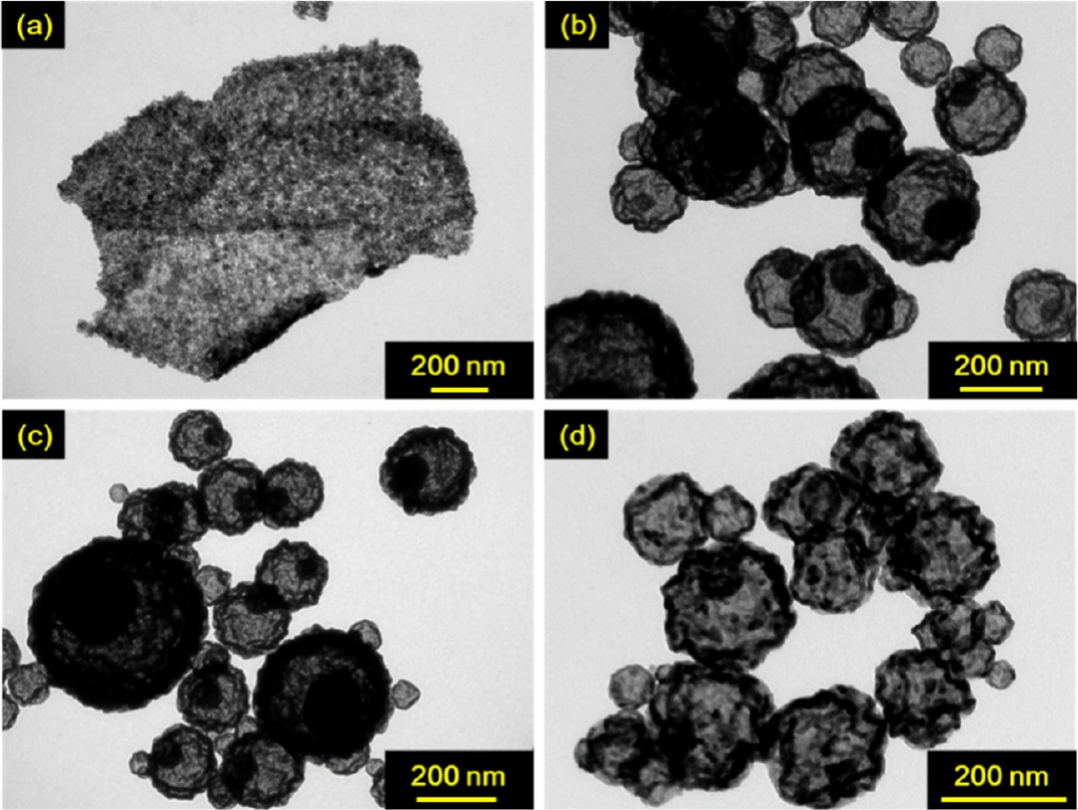
TEM images of (a) S-MCN-250–2-C, (b) as-synthesized S-MCN-600–2, (c) as-synthesized S-MCN-800–2, and (d) as-synthesized S-MCN-1000–2.

As shown in Figure 6a, the CeO_2_ composites synthesized at 250 °C, 400 °C, 600 °C, 800 °C, and 1000 °C exhibit weight losses of approximately 83%, 74%, 5%, 2%, and 2%, respectively. The significant mass loss observed for the S-MCN-250–2 sample is attributed to the high content of residual PVP and undecomposed cerium nitrate precursor. In contrast, at temperatures above 600 °C, the majority of the PVP template decomposes during spray pyrolysis, enabling the direct formation of yolk–shell structures.

**Figure 6:**
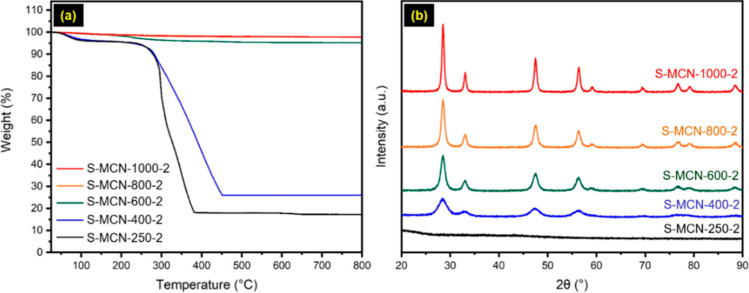
(a) Thermogravimetric curves and (b) XRD patterns of as-synthesized CeO_2_ particles produced at different furnace temperatures.

Figure 6b presents the XRD patterns of as-synthesized CeO_2_ particles at various furnace temperatures. The as-synthesized S-MCN-250–2 sample exhibits no distinct diffraction peaks, suggesting that the material remains largely amorphous at this low temperature. However, other samples exhibit distinct diffraction peaks corresponding FCC fluorite structure of CeO_2_. With increasing furnace temperature, the diffraction peaks become sharper and more intense, reflecting improved crystallinity and grain growth. The average crystallite size, estimated using Scherrer’s equation, increases from 3.9 nm at 400 °C to 7.0, 9.5, and 13.7 nm at 600 °C, 800 °C, and 1000 °C, respectively. These results indicate that higher furnace temperature promotes complete decomposition of precursors and enhances crystal growth. It should be noted that these values correspond to crystallite sizes calculated from XRD data and should therefore be distinguished from the larger particle dimensions observed in TEM images.

As shown in Figure S6, the N_2_ adsorption–desorption isotherm of the MCNs produced at 250 °C shows the H_3_-type hysteresis loops, characteristic of slit-shaped pores typically associated with layered or sheet-like structures, which is in close agreement with the TEM result. However, MCNs produced at temperatures above 400 °C exhibit H_2_-type loops, suggesting ink-bottle-shaped pores corresponding to their yolk–shell structures. The measured BET specific surface areas of S-MCN-250–2-C, S-MCN-400–2-C, as-synthesized S-MCN-600–2, as-synthesized S-MCN-800–2, and as-synthesized S-MCN-1000–2 samples are 120, 65, 74, 68, 38 m^2^/g, respectively (Table 1). The high specific surface area of S-MCN-250–2-C sample is attributed to its interconnected nanosheet aggregates, while the lower surface area of S-MCN-1000–2 results from significant primary particle size growth at elevated temperature.

#### 3.1.4 Tracking Yolk–Shell Formation in a Multi-Zone Furnace Setup

To gain deeper insight into the formation mechanism of yolk–shell-structured MCNs during spray pyrolysis, the custom-designed three-zone tube furnace was employed. This setup enabled the monitoring of particle evolution across controlled thermal stages along the reactor path. To determine appropriate temperature settings for each furnace zone, TGA-MS was conducted to investigate the thermal decomposition behavior of CeN up to 800 °C. Although the TGA-MS measurements were performed under an inert (argon) atmosphere to detect the released gaseous species,^50^ comparative TGA under air and argon showed nearly identical weight loss stages (Figure S7), confirming the reliability of the decomposition profile. As shown in Figure 7, the TGA-MS results revealed three main thermal events during CeN decomposition: dehydration below 200 °C with a weight loss of approximately 12%; decomposition of nitrate species and oxidation of anhydrous cerium (III) nitrate between 250 and 315 °C, accompanied by a significant weight loss of about 46%, and a gradual mass loss above 315 °C, indicating the crystallization of CeO_2_ and high thermal stability of the final oxide phase.^51,52^ Combined with the thermal behavior of the PVP template, these observations suggest that at 250 °C, only the drying process occurs; at 400 °C, CeN fully decomposes while PVP undergoes partial carbonization, leading to the formation of a carbon-CeO_2_ composite; and above 600 °C, CeO_2_ crystallizes and the residual carbon is predominantly combusted. Based on these observations, the zone temperatures of the multizone furnace were set to 250 °C, 400 °C, and 600 °C to facilitate a stepwise transformation: drying, composite formation, and carbon removal along with CeO_2_ crystallization.

**Figure 7:**
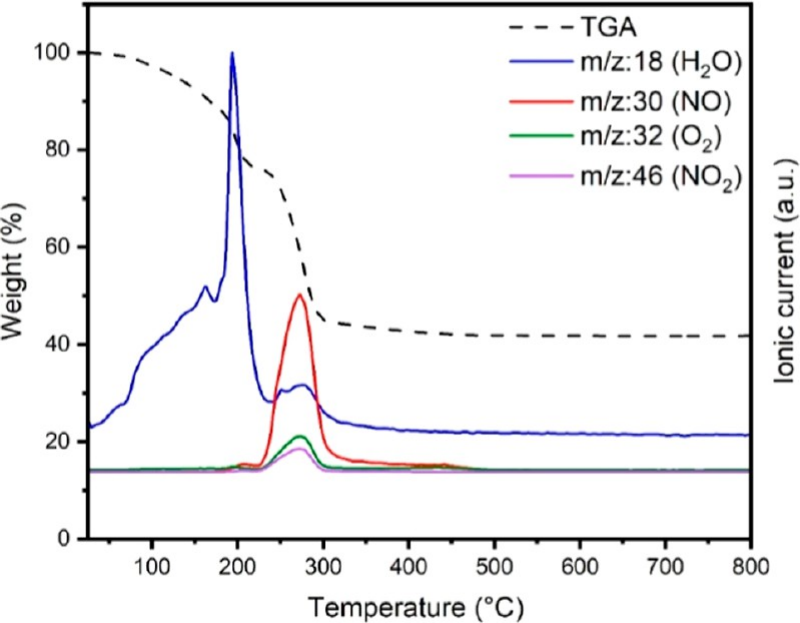
Simultaneous TGA/MS analysis of CeN decomposition in argon.

As illustrated in Scheme 1, the precursor solution with a fixed PVP-to-Ce mass ratio of 0.5 was atomized using the Topas nebulizer and passed through the three-zone tube furnace, with the individual zones set to 250 °C, 400 °C, and 600 °C, respectively. To investigate the stepwise transformation of the particles, samples were collected separately at the outlet of each temperature zone directly onto a filter.

**Scheme 1:**
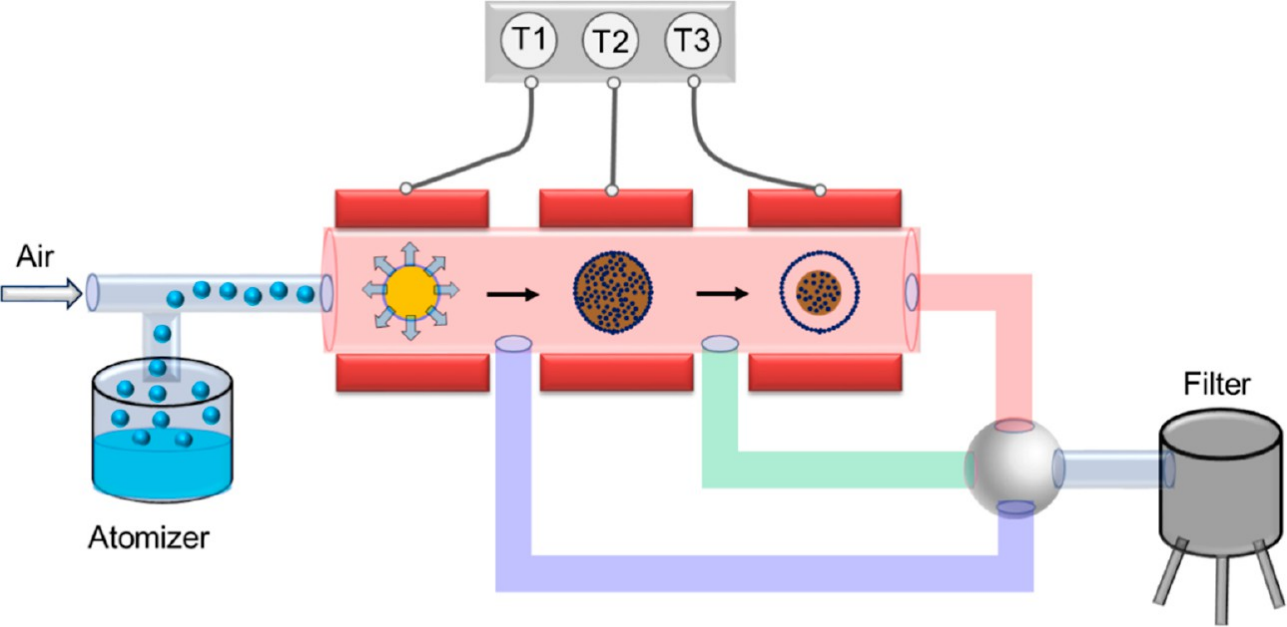
Schematic Representation of the Experimental Multi-Zone Tube Furnace Setup Used to Investigate the Stepwise Formation of Yolk–Shell-Structured MCNs During Spray Pyrolysis

Figure 8 shows TEM images of MCNs collected at different stages of the multizone furnace, both before and after calcination. For the as-synthesized samples, at 250 °C (Figure 8a), the particles are smooth, dense, and spherical, indicating only drying of the precursor droplets. At 400 °C (Figure 8b), internal porosity begins to develop due to the partial decomposition of CeN and carbonization of PVP, leading to the formation of composite particles consisting of semicrystalline cerium oxide intermixed with carbonaceous species. By 600 °C (Figure 8c), yolk–shell structures appear, indicating the crystallization of CeO_2_ and the combustion of carbon residues. After calcination, structural transformations are further enhanced. The calcined MZ-MCN-250–0.5-C (Figure 8d) collapses into porous aggregates, confirming structural instability. In contrast, MZ-MCN-400–0.5-C (Figure 8e) and MZ-MCN-600–0.5-C (Figure 8f) exhibit well-defined yolk–shell morphologies, demonstrating complete crystallization and the removal of residual carbon.

**Figure 8:**
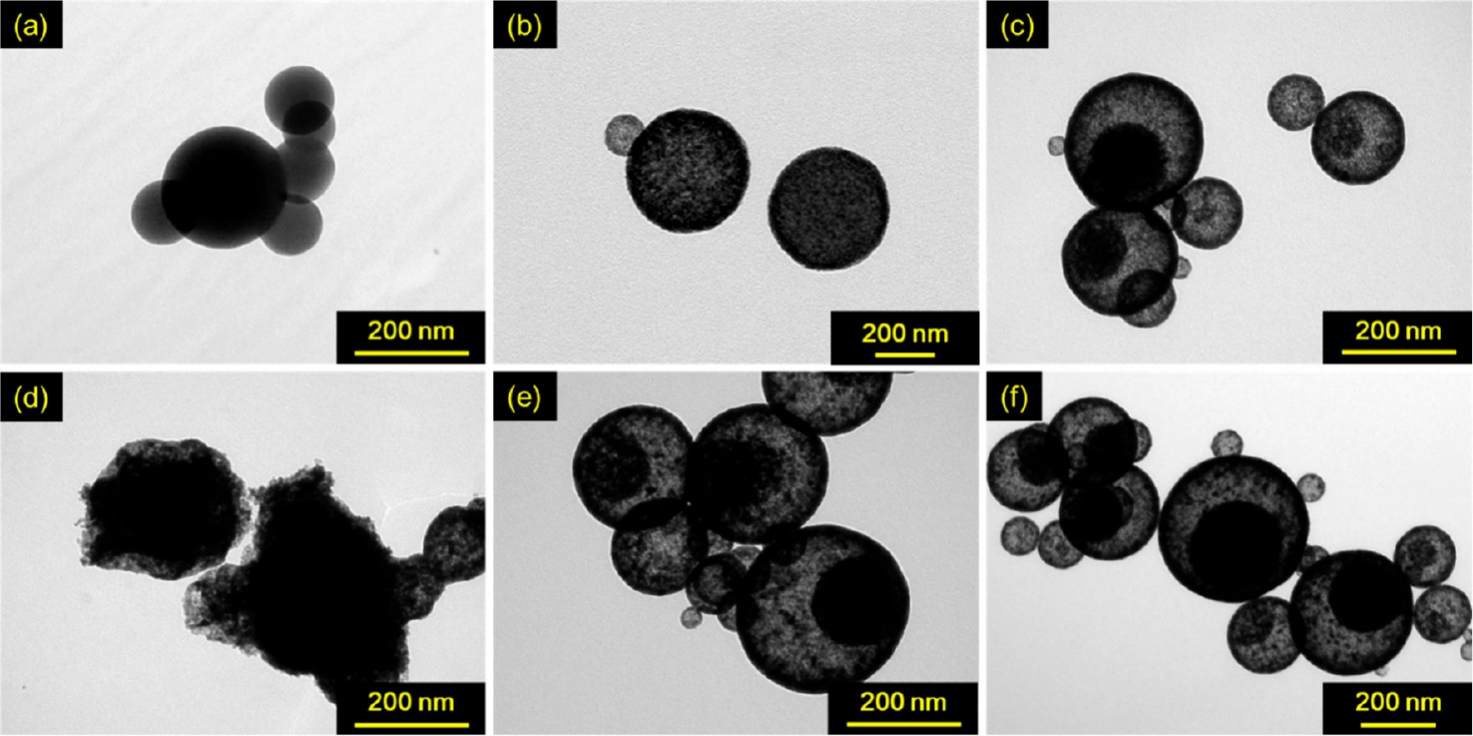
TEM images of (a) as-synthesized MZ-MCN-250–0.5, (b) as-synthesized MZ-MCN-400–0.5, (c) as-synthesized MZ-MCN-600–0.5, (d) MZ-MCN-250–0.5-C, (e) MZ-MCN-400–0.5-C, and (f) MZ-MCN-600–0.5-C samples.

TGA analysis shows that the as-synthesized MZ-MCN-y-0.5 composites prepared at 250 °C, 400 °C, and 600 °C exhibit weight losses of approximately 68%, 44%, and 8%, respectively (Figure 9a). The significant weight loss at lower temperatures is due to residual organics and undecomposed precursors, while a negligible loss observed at 600 °C confirms effective decomposition and direct yolk–shell formation.

**Figure 9:**
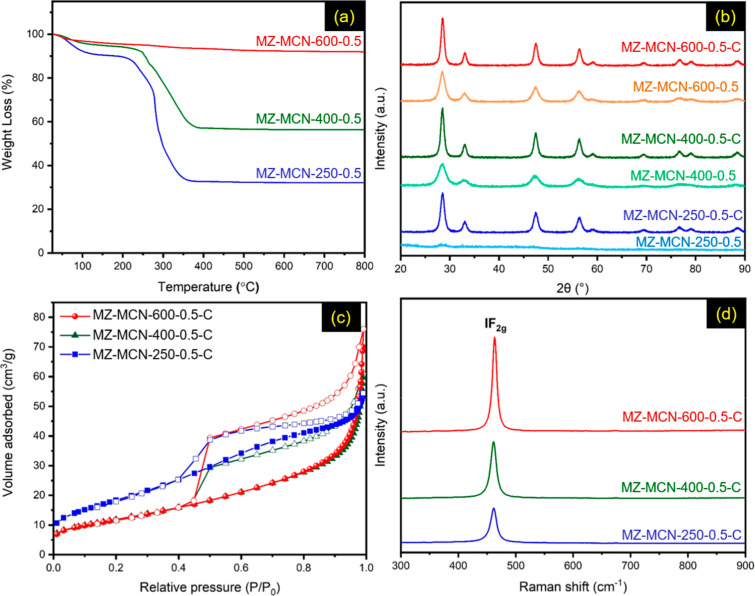
(a) Thermogravimetric curves of as-synthesized MZ-MCN-y-0.5 particles, (b) XRD patterns, (c) N_2_ adsorption–desorption isotherms, and (d) Raman spectra of MZ-MCN-y-0.5-C samples.

Figure 9b shows the XRD patterns of MZ-MCN-y-0.5 samples before and after calcination. The sample synthesized at 250 °C is mostly amorphous, while after calcination (MZ-MCN-250-0.5-C), distinct diffraction peaks appear, confirming the formation of crystalline CeO_2_ with an average crystallite size of 8 nm. At 400 °C, the as-synthesized sample shows weak and broad peaks corresponding to a small crystallite size of 4.2 nm. Upon calcination (MZ-MCN-400-0.5-C), the peaks become sharper, and the crystallite size increases to 9.8 nm. The 600 °C sample (MZ-MCN-600-0.5) already exhibits well-defined peaks (6.2 nm), suggesting partial crystallization during spray pyrolysis, which further improves upon calcination to 10.3 nm for MZ-MCN-600-0.5-C. The results confirm that increasing synthesis temperature and applying postcalcination both significantly enhance crystallinity and crystallite growth.

The N_2_ adsorption–desorption isotherms of the MZ-MCN-y-0.5-C samples show type IV curves with H_2_-type hysteresis loops (Figure 9c). The calculated BET surface areas are 68 m^2^/g for MZ-MCN-250–0.5-C, and 43 m^2^/g for both MZ-MCN-400–0.5-C and MZ-MCN-600–0.5-C. The higher SSA observed for the 250 °C sample can be attributed to its smaller crystallite size and the presence of loosely packed, porous structures, as also supported by the TEM image. Raman spectroscopy has been proven as an effective method to study the defects of CeO_2_ materials. As shown in Figure 9d, the MZ-MCN-600-C sample displays a sharp peak at 463 cm^–1^, corresponding to the F_2_g vibrational mode of fluorite-phase CeO_2_, which arises from the symmetric stretching of oxygen atoms around Ce^4+^ ions. However, this intense peak was red-shifted to 461.8 cm^–1^ and became broader and more asymmetric in the MZ-MCN-250-C and MZ-MCN-400-C samples. This red shift in the F_2_g mode has been reported to be typically associated with lattice expansion resulting from the incorporation of Ce^3+^ ions.^12^ The change in lattice parameter (Δα) relative to the Raman shift (Δω) can be estimated using the following eq (eq 4)­

4 $$\Delta \alpha =-\Delta {\omega \alpha }_{0}/3{\gamma \omega }_{0}$$

where α_0_ = 0.5411 nm and ω_0_ = 465 cm-1 for bulk CeO_2_, and γ is the Grüneisen constant (1.24). Based on this equation, the estimated lattice constants for MZ-MCN-600-C, MZ-MCN-400-C, and MZ-MCN-250-C samples are 5.417 Å, 5.421 Å, and 5.421 Å, respectively. Combined results from Raman and XRD suggest that the lattice parameter of CeO_2_ increases as the crystallite size decreases. This expansion is likely associated with the incorporation of Ce^3+^ ions into the lattice, which leads to a higher concentration of oxygen vacancies. Ce^3+^ ions have a larger ionic radius (1.034 Å) than that of the Ce^4+^ ions (0.92 Å). As the crystallite size decreases, the relative amount of Ce^3+^ increases, leading to more oxygen vacancies. This change in ionic composition and defect concentration causes the lattice to expand.

### 3.2 Formation Mechanism of MCNs

This study systematically investigates the influence of droplet size, furnace temperature, and PVP-to-Ce mass ratio on the morphology of MCNs produced by the spray pyrolysis process. The proposed formation mechanism is illustrated in Scheme 2. For the sample produced from small droplets at 250 °C, XRD and TGA/MS results show that the as-synthesized sample remains mostly amorphous with a high content of residual PVP and undecomposed CeN. This indicates that only the drying process occurs and the furnace temperature is insufficient to induce pyrolysis or precursor decomposition. In this regime, particle formation is primarily governed by solvent evaporation and diffusion-driven redistribution of components within the droplet. As the droplets pass through the heated zone, rapid solvent evaporation leads to a meniscus formation and capillary flow. According to the Stokes–Einstein eq (eq 5).^53,54^

5 $$D_{C}=k\frac{K_{B}T}{6\pi R_{C}\mu_{C}}$$

where *D*_C_ is the diffusion coefficient of the component (m^2^/g), *k* is the diffusion correction factor, *K*_B_ is the Boltzmann’s constant (1.38 × 10^23^ J/K), *T* is the temperature (K), μ_C_ is the solvent viscosity (kg/m·s), and *R*_c_ is the hydrodynamic radius of the diffusing species (*m*). Because the diffusion coefficient is inversely proportional to the component size, Ce^3+^ ions (with *R*_Ce_ ≈ 0.4 nm)^55^ diffuse over five times faster than PVP polymer chains (*M*_w_ = 10,000, *R*_PVP_ ≈ 2.16 nm)^56^ within the drying droplet. This disparity in diffusion rates causes Ce^3+^ ions to migrate rapidly toward the droplet surface, while the slower-diffusing PVP accumulates in the interior.^53,57^ As a result, a precursor structure resembling a core–shell configuration is formed. Upon calcination, the as-synthesized S-MCN-250 collapses into porous nanosheet aggregates due to structural instability arising from the high residual PVP content.

**Scheme 2:**
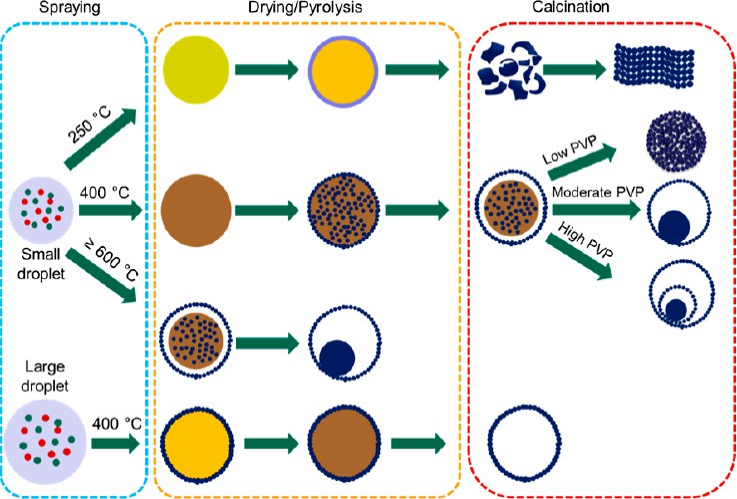
Schematic Illustration of the Formation of the Mesoporous Nanosheet, Yolk–Shell, and Hollow MCNs by Spray Pyrolysis

For the sample produced from small droplets at 400 °C with a moderate PVP-to-Ce mass ratio, spray pyrolysis leads to decomposition of CeN into CeO_2_, while the PVP undergoes partial carbonization, forming a CeO_2_-carbon composite. At this temperature, complete combustion of the carbon component does not occur, resulting in spherical solid particles without internal voids. Upon postcalcination, these composite particles gradually transform into yolk–shell-structured MCNs. Initially, combustion begins at the particle surface where oxygen access is sufficient, forming a stable crystalline CeO_2_ shell. However, limited oxygen diffusion to the particle core delays combustion of the internal carbon content, creating an intermediate core–shell (CeO_2_@CeO_2_–C) structure. As heating continues, the internal core shrinks due to carbon removal, while the rigid CeO_2_ shell maintains its form, consequently creating a single yolk–shell morphology. At high initial PVP concentrations, incomplete carbon removal in the core may lead to the formation of double-shelled yolk–shell structures. At synthesis temperatures above 600 °C, yolk–shell-structured MCNs formed directly during spray pyrolysis without requiring calcination. This indicates that the high thermal energy within the furnace is sufficient to completely decompose both the CeN precursor and the PVP. Consequently, the same structural evolution, typically observed during calcination, occurs spontaneously during the in-flight transformation process within the reactor. Further details on this transformation pathway are discussed in related studies.^28–32^

Interestingly, when larger droplets are used, the particles produced at 400 °C exhibit a hollow morphology. This phenomenon can be explained based on a model proposed by Lenggoro et al.^58^ In the case of small droplets (∼1.6 μm) produced by the Topas nebulizer, the short diffusion path allows for a relatively uniform solute concentration profile during solvent evaporation. This leads to simultaneous supersaturation throughout the droplet, yielding uniformly precipitated solid CeO_2_-carbon composite particles. In contrast, larger droplets (∼5.5 μm), generated using the Palas nebulizer, undergo slower evaporation, and the CeN precursor cannot fully diffuse back to the center. As a result, CeN accumulates near the surface while the droplet core remains rich in PVP. When the surface concentration of CeN reaches the critical supersaturation concentration while the core is still below equilibrium saturation, nucleation begins only at the surface, forming a shell. During subsequent calcination, the PVP in the core of particles is removed, giving rise to hollow-structured MCNs. It is worth mentioning that even in the case of large droplets, yolk–shell-structured MCNs could be directly produced at a high furnace temperature of 800 °C (as-synthesized L-MCN-800–0.5), rather than forming hollow particles (Figure S8). At elevated temperatures, higher thermal energy increases the diffusion rate of solutes (CeN) within the droplet, as described by the Stokes–Einstein relation (eq 5). At 800 °C, the increased *D*_C_ facilitates more uniform precipitation of CeN throughout the droplet and accelerates both precursor decomposition and PVP carbonization, resulting in the formation of CeO_2_ particles with a yolk–shell structure.

### 3.3 Surface Characterization and Functionalization with Thiolated Dextran

The isoelectric point (pI) was determined from zeta potential measurements as a function of pH and was found to range from 4.7 to 5.8 for all synthesized MCNs (Figure 10a). Below the pI, the surface carries a positive charge due to protonation of hydroxyl groups (Ce–OH_2_^+^), whereas above this value, deprotonation (Ce–O^–^) results in a negatively charged surface. These pI values are consistent with those previously reported for porous CeO_2_ obtained by spray pyrolysis (∼5.2–5.7).^12^

**Figure 10:**
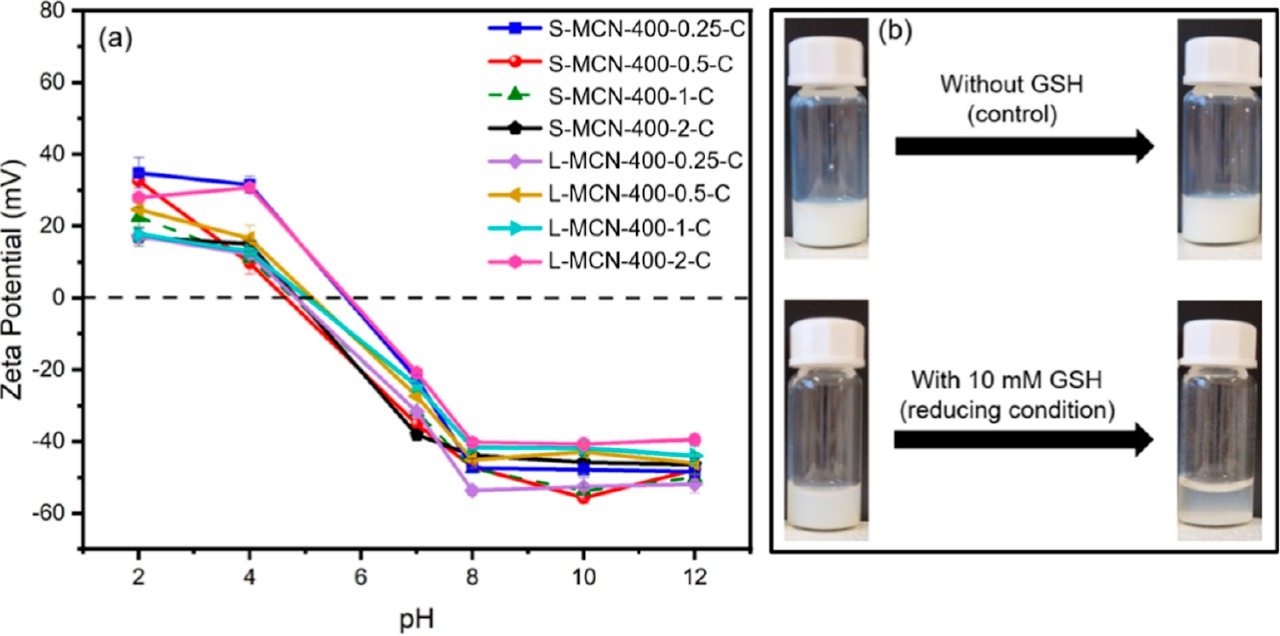
(a) Zeta potential values of MCN samples used for loading and biological experiments as a function of pH in deionized water, and (b) influence of glutathione on the stability of dextran-derivative-coated MCN suspensions at pH 7.4 in less than 1 min.

To evaluate the redox-responsiveness of the thiolated-dextran-functionalized MCNs, we monitored their colloidal stability under physiological and reducing conditions (Figure 10b). The MCNs modified with Dex6-SH were dispersed in aqueous medium (pH 7.4) at a high concentration (10 g·L^–1^), and two conditions were evaluated: without and with 10 mM glutathione (GSH), mimicking extracellular and intracellular redox environments, respectively. As shown in Figure 10b, the Dex6-SH-modified MCNs remained stably dispersed in the absence of reducing agent (GSH), with no visible aggregation or sedimentation. In contrast, the addition of glutathione induced immediate sedimentation, resulting from the cleavage of disulfide bonds linking the dextran to the MCNs surface. This bond rupture caused polymer detachment and, consequently, particle destabilization. These results confirm the successful surface functionalization of MCNs with thiolated-dextran and demonstrate their redox-responsiveness. Disulfide bonds are known to be stable in the extracellular environment, where glutathione concentrations are low (2–20 μM), but are readily cleaved intracellularly, where glutathione levels are significantly higher (∼10 mM),^59^ making this system highly relevant for controlled-release drug delivery applications.

### 3.4 Biocompatibility and Loading Capabilities

#### 3.4.1 Cellular Toxicity of MCNs

The evaluation of the biocompatibility of the synthesized MCNs was conducted by analyzing their impact on the viability of HEK293 cells. Cell viability was measured following a 24 h incubation with different concentrations of nanoparticles resuspended in PBS and diluted in culture media using the colorimetric CCK-8 assay. As shown in Figure 11a–h, the MCNs exhibited dose-dependent cytotoxicity. S-MCN-400–1-C and S-MCN-400–2-C did not significantly affect cell viability across the tested concentration range. In contrast, S-MCN-400–0.25-C affected cell viability at concentrations above 50 μg/mL, while S-MCN-400–0.5-C caused a noticeable decrease only at concentrations exceeding 600 μg/mL. L-MCN-400–0.25-C and L-MCN-400–0.5-C reduced viability at concentrations exceeding 100 μg/mL, whereas L-MCN-400–1-C and L-MCN-400–2-C exhibited the highest cytotoxicity, with significant viability loss observed at concentrations as low as 10 μg/mL. The observed differences may be attributed to variations in particle morphology, surface characteristics, or residual species originating from the synthesis process. Importantly, all tested MCNs maintained cell viability above 80% at 100 μg/mL, the concentration used in subsequent biological assays in this study. This indicates that, despite varying levels of cytotoxicity at higher doses, the nanoparticles exhibit acceptable biocompatibility under the conditions relevant to this study.

**Figure 11:**
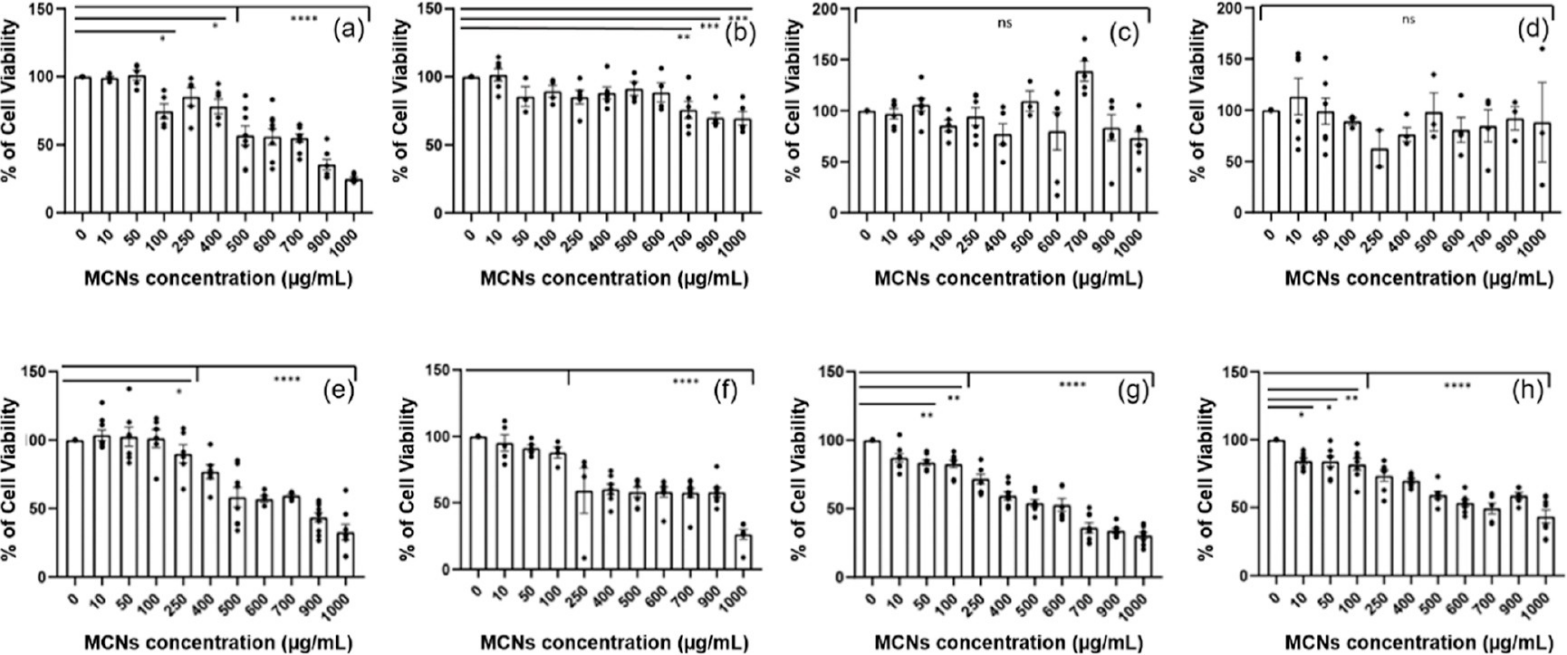
Cellular toxicity of (a) S-MCN-400–0.25-C, (b) S-MCN-400–0.5-C, (c) S-MCN-400–1-C, (d) S-MCN-400–2-C particles, (e) L-MCN-400–0.25-C, (f) L-MCN-400–0.5-C, (g) L-MCN-400–1-C, and (h) L-MCN-400–2-C particles. HEK293 cells were treated for 24 h with varying concentrations (from 10 μg/mL to 1000 μg/mL) of the indicated nanoparticles and cell viability was assessed using the CCK-8 assay. Values were normalized to untreated cells (0 μg/mL) and data are shown as mean ± SEM. Statistical analysis was performed using one-way ANOVA followed with a Turkey test for *p* value evaluation (ns for not significant, * for *p* < 0.05, ** for *p* < 0.01, *** for *p* < 0.001 and **** for *p* < 0.0001).

#### 3.4.2 Probe Loading Efficiency

Negatively charged MCNs were exposed to rhodamine 6G (Rh6G), a small (a few nanometers) cationic fluorescent molecule. Their textural properties, specifically surface area, pore volume, and pore size distribution, facilitated efficient encapsulation of the dye within the porous network. Fluorescence microscopy of the Rh6G-loaded particles (Figure S9) showed a strong red emission, confirming successful incorporation of the probe into the particles. In contrast, unmodified CeO_2_ particles exhibited no detectable fluorescence.

We also investigated the loading of Rh6G into the CeO_2_ samples. As a small molecular probe, Rh6G can diffuse into the internal volume of the particles. Moreover, Rh6G is positively charged, while MCNs are negatively charged (below −20 mV) under the loading conditions, promoting electrostatic interactions that facilitate Rh6G entrapment. To confirm this, the zeta potential of Rh6G-loaded CeO_2_ particles was measured and found to be approximately +20 mV, supporting this hypothesis. The Rh6G loading efficiencies for the yolk–shell-structured MCNs S-MCN-400–0.5-C, S-MCN-400–1-C, and S-MCN-400–2-C were approximately 10%, 13%, and 7%, respectively. A quantitative correlation between the total void volume, calculated from TEM-derived geometrical parameters using eq 3, and Rh6G loading efficiency is presented in Figure 12. For these investigated yolk–shell MCNs, the loading efficiency increases with increasing void volume, showing a clear structure–function relationship (*R* ^2^ = 0.82). Since Rh6G loading was measured for the whole powder sample, whereas void volume was estimated from well-defined yolk–shell particles in TEM images, the observed correlation should be considered a semiquantitative structure–function trend. This correlation suggests that the accessible internal cavity contributes substantially to probe encapsulation. Furthermore, L-MCN-400–0.5-C, which features a hollow structure and was synthesized using larger droplets, exhibited a significantly higher loading efficiency of 42%. This enhanced capacity is likely due to its larger internal free volume, allowing for greater dye uptake compared to yolk–shell counterparts. Therefore, the observed relationship should be interpreted as a structure-dependent trend within the investigated MCN samples. However, this relationship should not be interpreted as a universal correlation for all CeO_2_ morphologies, as pore accessibility, surface charge, and dye–surface interactions may also contribute to the loading behavior. These findings highlight how structural differences, influenced by synthesis parameters such as droplet size and PVP content, affect the functional loading capabilities of MCNs.

**Figure 12:**
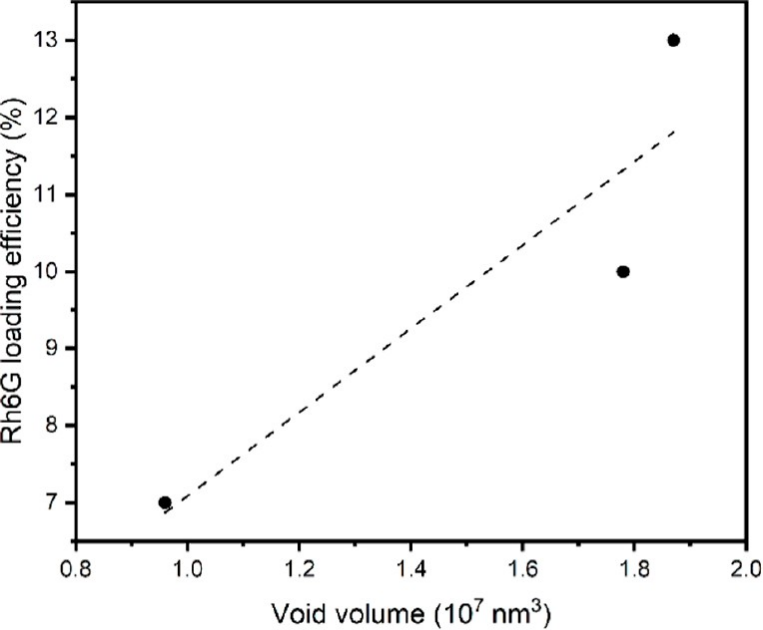
Rh6G loading efficiency as a function of total void volume, calculated using eq 3, for yolk–shell-structured MCNs.

### 3.5 Drug Delivery Potential and Functional Assays

#### 3.5.1 G418-Induced Readthrough Assay (Luciferase)

To evaluate the potential of MCNs for enhancing the functional delivery of therapeutics, we assessed their effect on G418-induced translational readthrough using an in vitro luciferase reporter assay. The assay principle is schematically illustrated in Figure 13A. U2OS cells stably transfected with a FLuc-int UGA construct were incubated with increasing concentrations of G418, either alone or in combination with different nanoparticles.^60^ Experiments were performed independently three times, and the corresponding results are shown in Figure 13B. In all three experiments, the presence of G418 induced a dose-dependent increase in luciferase activity, reflecting readthrough of the premature stop codon. When G418 was preincubated with 100 μg/mL of nanoparticles, a higher luciferase activity was observed at doses above 25–50 μg/mL as compared to G418 alone. This effect was particularly pronounced for L-MCN-400–1-C, which consistently yielded the highest luciferase signals, suggesting superior readthrough facilitation. Although the amplitude of the response varied between independent experiments, the overall dose–response profile remained consistently greater in the presence of nanoparticles than the G418 alone condition.

**Figure 13:**
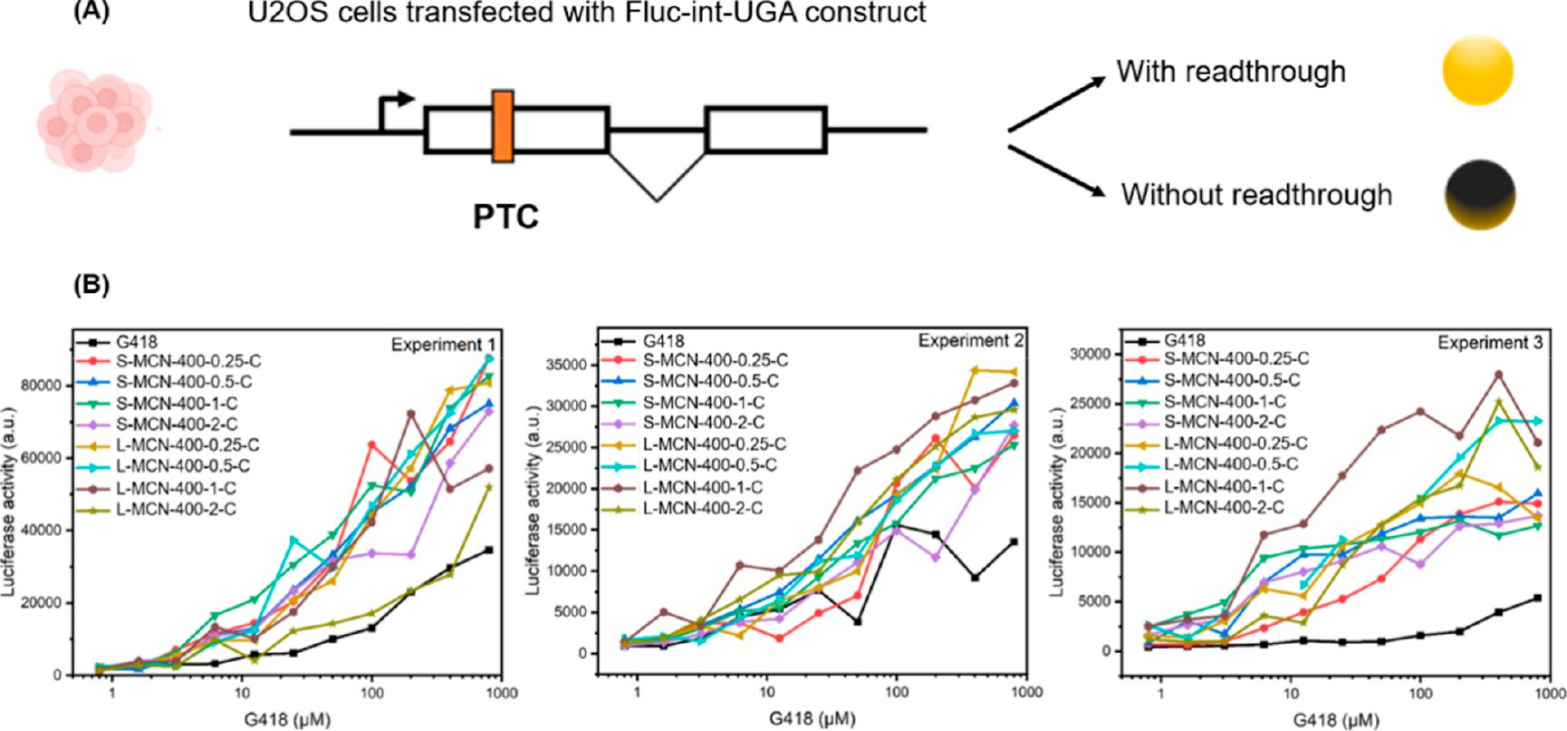
Effect of MCNs on G418-induced readthrough. (A) Schematic representation of the in vitro luciferase readthrough assay, (B) MCNs enhance the readthrough activity of G418. U2OS cells were incubated with increasing doses of G418, either complexed or not with nanoparticles, prior to measuring luciferase activity as a readout of readthrough efficiency. Results from three independent experiments are shown.

#### 3.5.2 G418-Induced Rescue of CFTR-R553X Function (MTECC)

The transepithelial resistance of untreated 16HBEo-WT cells was measured using a modified Ussing chamber technique called Multi Transepithelial Current Clamp (MTECC). Cells were grown on filters under liquid–liquid conditions to record the electrical parameters of 24 filters simultaneously. CFTR function was assessed by activating the channel with the agonists Forskolin and IBMX, as well as the potentiator VX-770. CFTR was then specifically inhibited by Inh172, and the equivalent current (Ieq) was determined. In 16HBEo- cells expressing WT CFTR, the Ieq response measured under Inh172 addition was of −30 ± 1.1 μA/cm2 (*n* = 24) in basal conditions. Under the same condition, 16HBEo- R553X cells did not show basal activity (Figure 14). Cells were treated with increasing amounts of G418 (from 50 μg/mL to 400 μg/mL) for 48 h. To maximize CFTR protein and mRNA amounts, cells were incubated with CFTR correctors VX-445 (3 μM) and VX-809 (3 μM) and nonsense-mediated decay (NMD) inhibitor SMG1 (0.5 μM). In the presence of 400 μg/mL of G418, transepithelial electrical resistance (*R*_t_) dropped under 200 Ohms, indicating a toxic effect at this concentration. In response to increasing amounts of G418 comprised between 50 μg/mL and 300 μg/mL, the Ieq current measured after CFTR inhibition showed a dose-dependent increase that became statistically significant at concentration 200 μg/mL and 300 μg/mL. The addition of L-MCN-400–1-C or S-MCN-400–2-C that highly promoted readthrough activity (Figure 13) was tested. These MCNs alone did not affect *R*_t_ or Ieq (Figure 14). Combining G418 and L-MCN-400–1-C or S-MCN-400–2-C significantly increased Ieq at 100 μg/mL, 200 μg/mL and 300 μg/mL. These results indicate that L-MCN-400–1-C and S-MCN-400–2-C slightly increased readthrough of CFTR-R553X in the presence of 100 μg/mL of G418, even though the effect remained limited.

**Figure 14:**
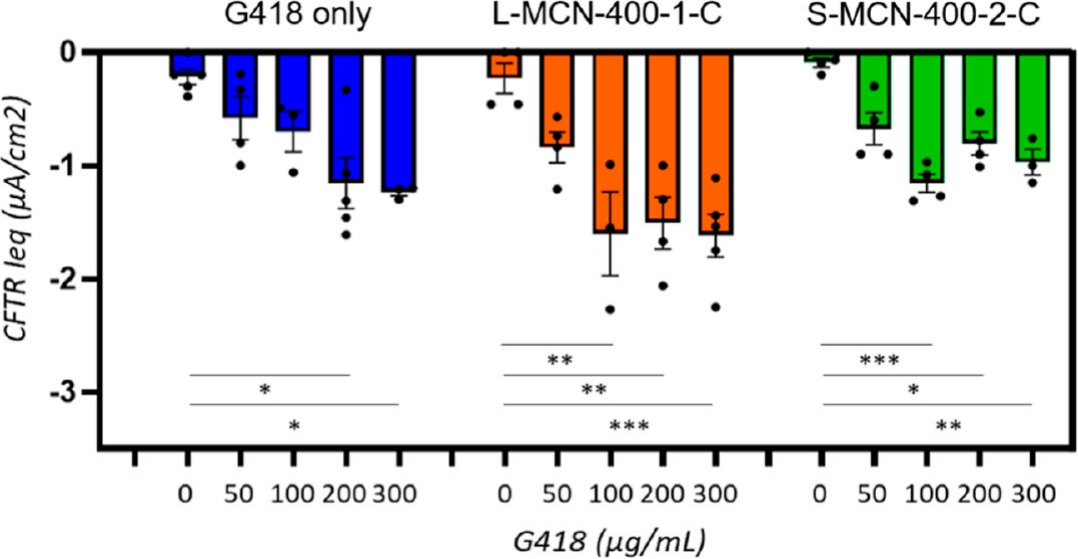
Effect of nanoparticles on the rescue of CFTR-R553X activity. CFTR activity was measured using the MTECC procedure in polarized 16HBEo- R553X cells. Cells were treated 48 h with increasing amounts of G418, from 50 μg/mL to 300 μg/mL alone or complexed with 100 μg/mL of L-MCN-400–1-C or S-MCN-400–2-C. To maximize rescued CFTR amounts, cells were treated with CFTR modulators and NMD inhibitor. Data are shown as mean ± SEM and statistical analysis was performed using one-way ANOVA followed with a Turkey test for *p* value evaluation (* for *p* < 0.05, ** for *p* < 0.01 and *** for *p* < 0.001).

## 4 Conclusion

In this study, we developed a versatile gas-phase approach for the synthesis of morphology-tailored MCNs via spray pyrolysis, enabling the controlled fabrication of solid, hollow, and yolk–shell architectures. In contrast to conventional solution-based methods that rely on multistep processing and sacrificial layers, our spray pyrolysis approach provides a rapid and straightforward synthesis route for synthesizing MCNs with adjustable morphologies. This gas-phase process avoids hazardous etching agents, enables direct collection of dry powders, and minimizes aggregation by treating each atomized droplet as an individual microreactor. By systematically adjusting the precursor composition, droplet size, and thermal profile, we achieved fine-tuning of internal cavity size, shell porosity, and particle morphology. A custom-built multizone furnace provided mechanistic insight into morphology evolution through monitoring of particle formation across controlled thermal stages along the reactor path. Furthermore, surface functionalization with thiolated dextran imparted redox-responsive behavior to the MCNs, enabling disulfide bond cleavage under intracellular-mimicking conditions, as demonstrated by glutathione-triggered particle destabilization. Zeta potential and Rhodamine 6G loading studies further revealed the influence of particle morphology on surface charge and cargo encapsulation efficiency. Biological evaluation confirmed the biocompatibility of MCNs in HEK293 cells at relevant concentrations. Functional assays in mammalian cell models demonstrated that MCNs significantly enhanced the performance of the aminoglycoside G418 in promoting translational readthrough in U2OS luciferase reporter cells and partially restored CFTR-R553X activity in epithelial monolayers under optimized conditions. The current work focuses on morphology-controlled synthesis, pore and void formation, redox-responsive surface functionalization, loading capability, and biological delivery performance. A systematic investigation of quantitative defect chemistry, including characterization of Ce^3+^ concentration, oxygen vacancies, and antioxidant activity, will be addressed in future work. Altogether, this work establishes a continuous gas-phase synthesis route for the synthesis of engineered CeO_2_ nanostructures and highlights their potential as redox-sensitive delivery vehicles for advanced biomedical applications, including gene rescue therapies.

## Supplementary Material



## Notes

Multizone furnace configuration; void-volume calculation method for yolk–shell MCNs; SEM images of S-MCN-400-z-C samples; pore-size distributions and XRD patterns with CeO_2_ reference pattern; droplet-size distributions of Topas and Palas atomizers; TGA curves of Palas-derived MCNs and cerium nitrate precursor; pore-size distributions of L-MCN-400-z-C samples; N_2_ adsorption–desorption isotherms of temperature-series MCNs; TEM image of as-synthesized L-MCN-800–0.5; fluorescence images of Rh6G-loaded MCNs (PDF)
