# Supplementary material for: Spray-Pyrolyzed Hollow and Yolk–Shell CeO2 Nanocarriers with Tunable Structure for Redox-Responsive Delivery and Gene Rescue Applications
Source: ACS Appl Mater Interfaces. 2026 Jun 26;18(27):38266–83. doi: 10.1021/acsami.6c07497 (PMC13383284; doi:10.1021/acsami.6c07497)
Supplement: Supplementary file 1 [file am6c07497_si_001.pdf]

Supporting Information

Spray-Pyrolyzed Hollow and Yolk-Shell CeO_2 Nanocarriers with Tunable Structure for Redox-Responsive Delivery and Gene Rescue Applications

Jalal Poostforooshan[1]*, Cléa Chesneau[2], Laurent Michely[2], Madara Dias Wickramanayaka[3], Iwona Pranke[3], Patrick Balaguer[4], Abdelhay Boulahtouf[4], Fabrice Lejeune[5], Benedikt Eberhardt[1], Alexandre Hinzpeter[3], Sabrina Belbekhouche[2]*, Alfred P. Weber[1]

[1]Institute of Particle Technology, Clausthal University of Technology, 38678 Clausthal-Zellerfeld, Germany

[2]Univ Paris Est Creteil, CNRS, Institut de Chimie et des Matériaux Paris-Est (ICMPE), UMR 7182, 2 Rue Henri Dunant, 94320 Thiais, France

[3]Université Paris Cité, CNRS, UMR8253, INSERM, U1151, Institut Necker Enfants Malades (INEM), F-75015 Paris, France

[4]Institut de Recherche en Cancérologie de Montpellier (IRCM), Inserm, U1194, Université de Montpellier, Institut régional du Cancer de Montpellier (ICM), 34090 Montpellier, France

[5]Univ. Lille, Inserm, CHU Lille, CNRS, U1366-UMR9020, CRCLille, Cancer Research Center of Lille, F-59000 Lille, France

Corresponding Authors

*Jalal Poostforooshan
Institute of Particle Technology, Clausthal University of Technology, 38678 Clausthal-Zellerfeld, Germany
Phone: +49 5323723574; Fax: +49 5323722830.
Email: jalal.poostforooshan@tu-clausthal.de

*Sabrina Belbekhouche
Université Paris Est Créteil, ICMPE (UMR7182), CNRS, UPEC, F-94320 Thiais, France
Phone: +33 (0)1 49 78 11 49; Fax: +33 (0)1 49 78 12 08.
Email: belbekhouche@icmpe.cnrs.fr

S1. Multi-Zone Furnace Configuration

The custom-built multi-zone furnace was constructed using a stainless steel V4A round tube with a total length of 1000 mm, an outer diameter of 30 mm, and an inner diameter of 26 mm. The reactor was divided into three independent controlled heating zones: the first zone (300 mm) was maintained at 250 °C, while the second and third zones (each 350 mm) were set at 400 °C and 600 °C, respectively. Each zone was equipped with a HORST heating tape, a dedicated temperature controller, and a NiCr-Ni thermocouple sensor. For thermal insulation and safety, HORST staple fiber tape, rated for temperatures up to 1000 °C, was used to wrap the heated sections. This setup allowed for a controlled temperature gradient along the tube, enabling the systematic study of particle evolution during aerosol passage through varying thermal environments.

S2. Void Volume Calculation Method

The void volumes inside the yolk-shell-structured MCNs were calculated based on the outer diameter of the particles, shell thicknesses, and yolk diameters measured from TEM images using ImageJ software. The geometrical parameters used for the void-volume calculations are summarized in Table S1.

Table S1. Geometrical parameters extracted from TEM images and used for void-volume calculation of yolk-shell MCNs.

Sample ID	D (nm)	S (nm)	Y (nm)	Additional Parameter(s)
S-MCN-400-0.5-C	403	21	235	–
S-MCN-400-1-C	390	22	179	–
S-MCN-400-2-C	303	S_1 = 14 (inner), S_2 = 16 (outer)	64	Z = 135 (outer diameter of inner shell)

where D is the outer diameter of the well-defined yolk-shell particle, S is the shell thickness, and Y is the yolk diameter, all determined from TEM images.

For single-shell particles (S-MCN-400-0.5-C, S-MCN-400-1-C), the void volume was calculated using eq S1:

$$V = (4/3)\cdot\pi\cdot[(D/2 - S)^3 - (Y/2)^3] \qquad \text{(S1)}$$

For the double-shell particle (S-MCN-400-2-C), the total void volume was computed using eq S2:

$$V_{total} = V_1 + V_2 \qquad \text{(S2)}$$

where V_1 is the void volume between the yolk and the inner shell, and V_2 is the void volume between the inner shell and the outer shell.

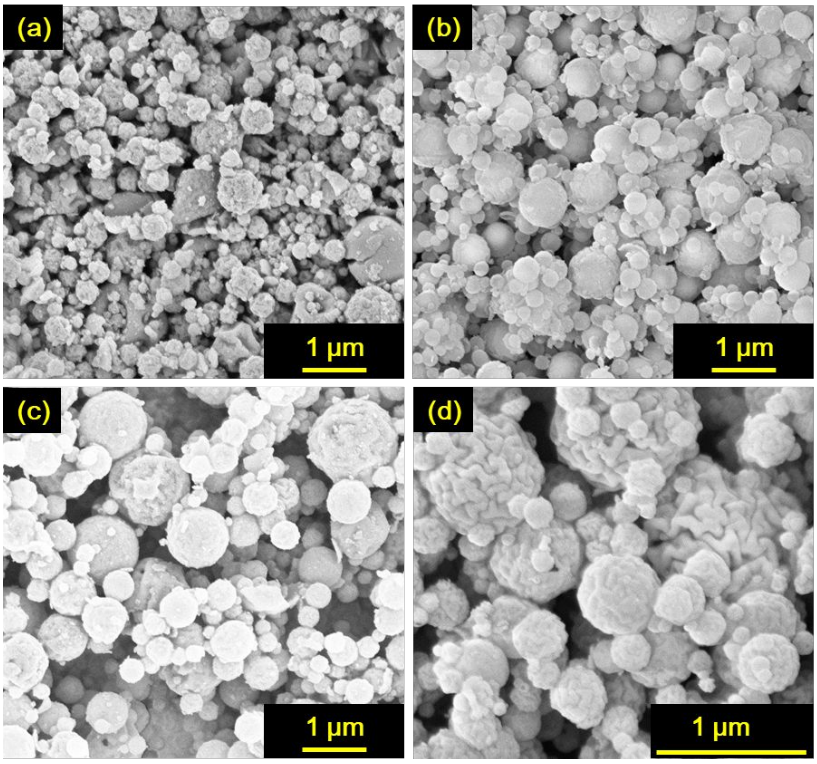

Figure S1. SEM images of (a) S-MCN-400-0.25-C, (b) S-MCN-400-0.5-C, (c) S-MCN-400-1-C, and (d) S-MCN-400-2-C particles prepared using spray pyrolysis.

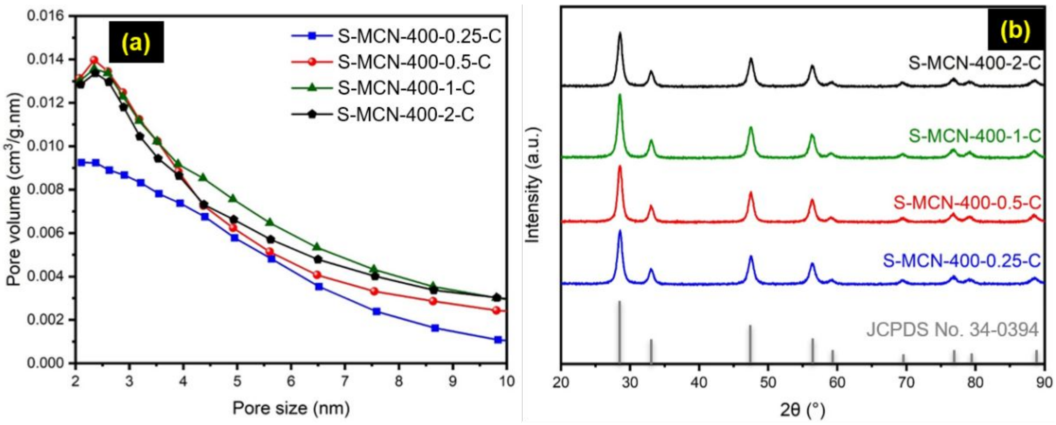

Figure S2. (a) Pore size distribution and (b) XRD patterns of S-MCN-400-z-C samples. The vertical bars in panel (b) indicate the standard diffraction pattern of cubic fluorite CeO_2 according to JCPDS No. 34-0394.

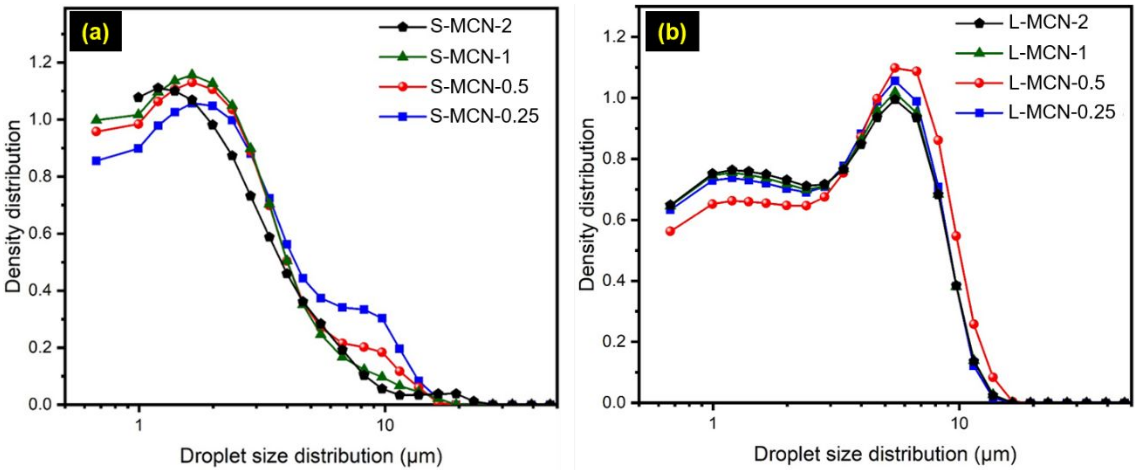

Figure S3. Droplet size distributions produced by (a) Topas ATM 220 and (b) Palas AGK-2000 atomizers.

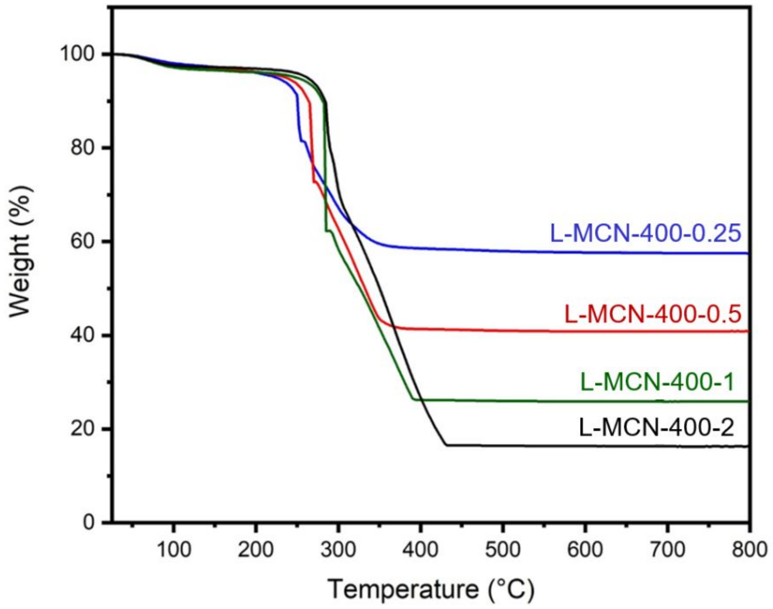

Figure S4. Thermogravimetric analysis (TGA) curves of as-synthesized L-MCN-400-z samples prepared using a Palas atomizer at varying PVP-to-Ce mass ratios.

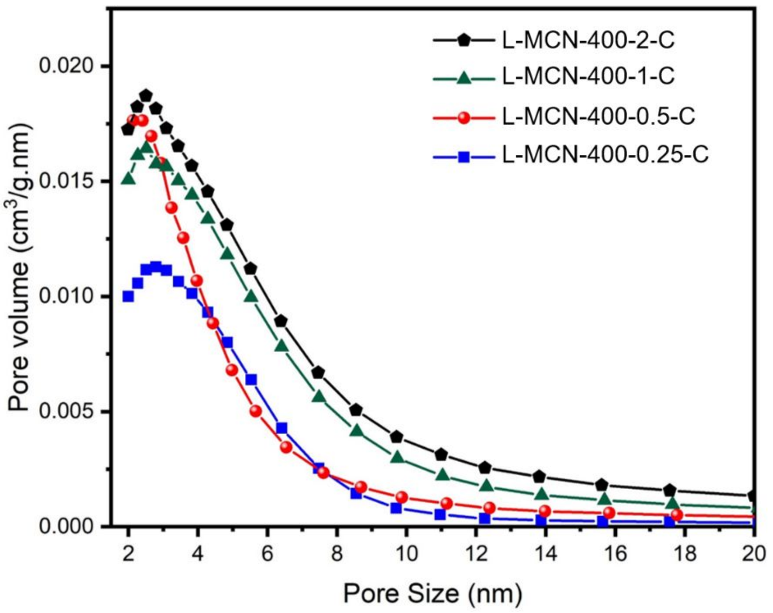

Figure S5. Pore size distribution of L-MCN-400-z-C samples.

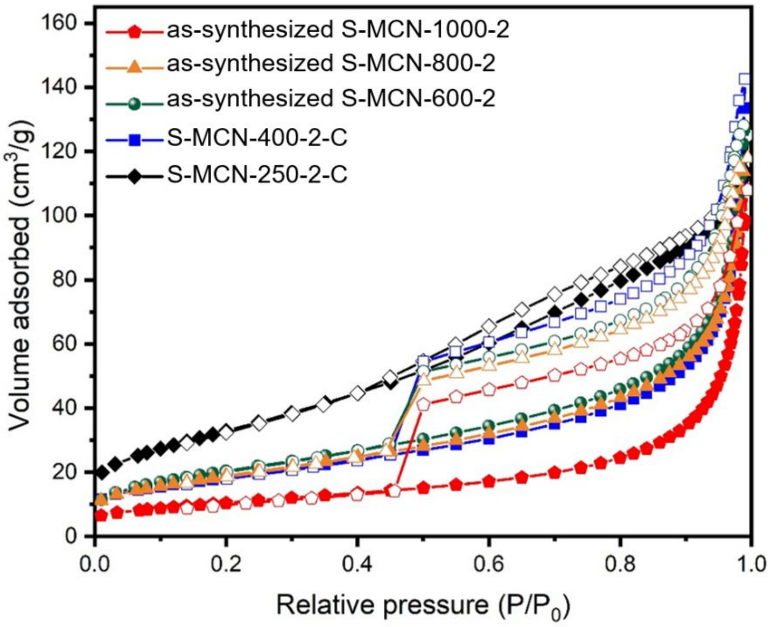

Figure S6. N_2 adsorption-desorption isotherms of MCNs produced at different furnace temperatures.

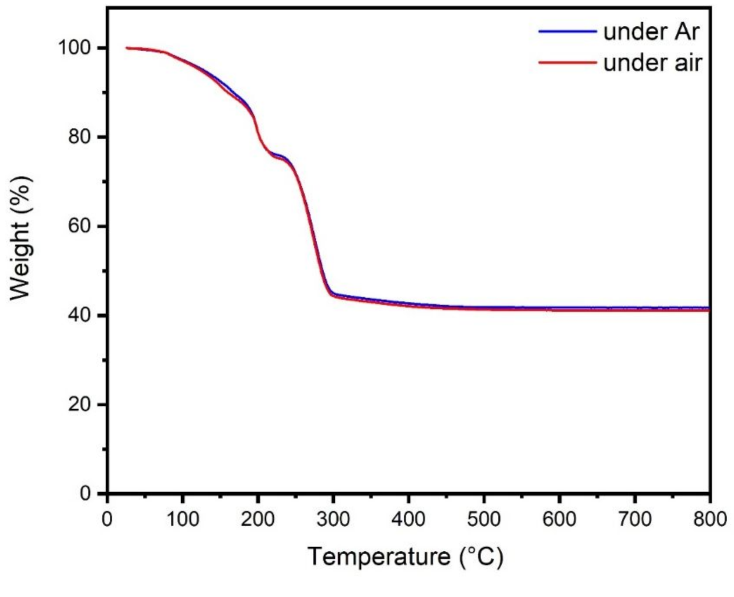

Figure S7. Thermogravimetric curves of the $Ce(NO_3)_3 \cdot 6H_2O$ under Ar and air atmospheres.

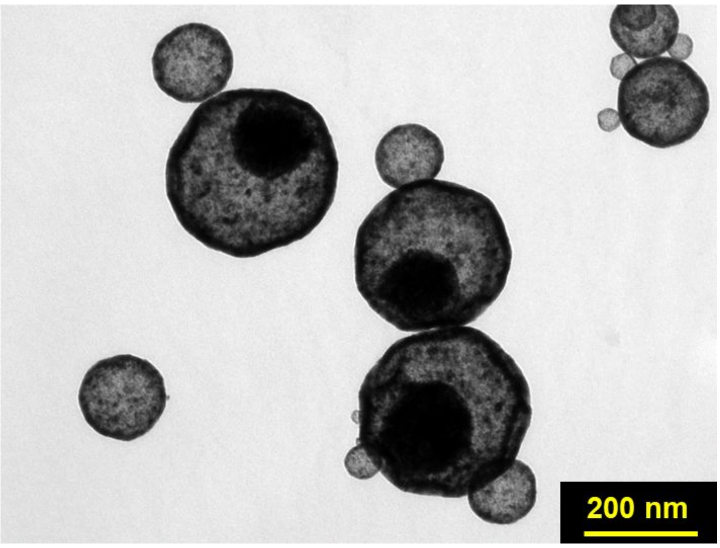

Figure S8. TEM image of as-synthesized L-MCN-800-0.5.

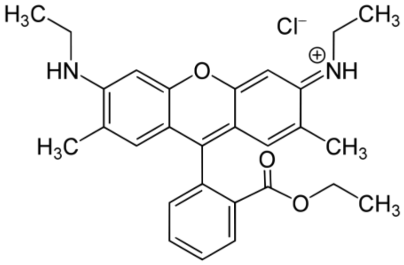

λ = 525 nm
ε_{exp} = 96012 L.mol^{-1}.cm^{-1}

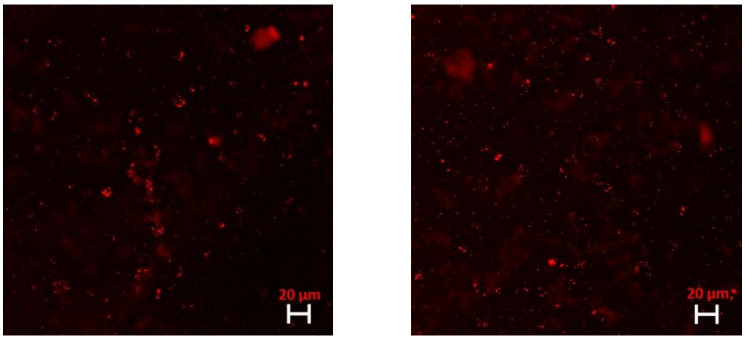

Figure S9. Fluorescence images of MCNs loaded with rhodamine 6G (Rh6G).
